# *In silico* interaction analysis of selected natural compounds with bacteriophage-encoded hyaluronate lyase from *Streptococcus pyogenes*

**DOI:** 10.3389/fmed.2026.1709023

**Published:** 2026-02-16

**Authors:** Samia S. Alkhalil

**Affiliations:** Department of Medical Laboratory Sciences, College of Applied Medical Sciences, Shaqra University, Alquwayiyah, Riyadh, Saudi Arabia

**Keywords:** bacteriophage, hyaluronate lyase, molecular docking, molecular dynamics, natural compounds, *Streptococcus pyogenes*

## Abstract

**Introduction:**

The rising antibiotic resistance of *Streptococcus pyogenes* necessitates alternative anti-virulence strategies. Bacteriophage-encoded hyaluronate lyase (HylP2), a key virulence factor that promotes bacterial dissemination by degrading host extracellular matrix components, represents an attractive therapeutic target.

**Methods:**

In this study, an integrated *in silico* approach was employed to identify potential HylP2 inhibitors from a library of 118 bioactive natural compounds. Following protocol validation through redocking of ascorbic acid (RMSD = 1.897 Å), virtual screening, ADMET prediction, molecular dynamics (MD) simulations, and per-residue energy decomposition analyses were performed.

**Results:**

Violacein (−7.7 kcal/mol), sulfangolid C (−7.427 kcal/mol), chlorotonil A (−7.4 kcal/mol), xiamycin (−7.3 kcal/mol), and kulkenon (−7.1 kcal/ mol) were identified as the most potent binders. ADMET analysis confirmed that these leads possess favorable pharmacokinetic properties and compliance with Lipinski’s Rule of Five. Subsequent 100-ns molecular dynamics (MD) simulations and per-residue energy decomposition revealed that violacein, xiamycin, and kulkenon formed stable, compact complexes by “trapping” catalytic residues Arg279 and Tyr264.

**Conclusion:**

These findings suggest that these natural product scaffolds are promising anti-virulence leads that may limit *S. pyogenes* tissue invasion while minimizing selective pressure for resistance development.

## 1 Introduction

*Streptococcus pyogenes* is a clinically significant human pathogen responsible for a wide spectrum of diseases, ranging from mild skin and throat infections to severe, life-threatening conditions such as sepsis, streptococcal toxic shock syndrome, scarlet fever, and necrotizing fasciitis (1). The Gram-positive, *β*-hemolytic bacterium, also known as group A streptococci; GAS, commonly colonizes the throat, vaginal mucosa, and anorectal region (2). The global prevalence of *S. pyogenes* infections varies depending on the clinical manifestations and geographic factors. Infection involves multiple factors, including intricate interactions between host immune defenses and bacterial virulence determinants that collectively affect disease pathophysiology (3).

Bacteriophages are the most abundant viruses in the human microbiome and play a crucial role in maintaining or disrupting microbial ecological balance. They influence human health both directly through bacterial predation, and indirectly, by modulating host immune responses and metabolic processes (4). Interactions between bacteriophages and their bacterial hosts are central to the evolution and dissemination of virulence traits. In *S. pyogenes*, numerous toxins and virulence factors are bacteriophage-encoded, and phage-derived sequences constitute an integral component of the GAS genome (5–7). Among these factors, bacteriophage-encoded hyaluronidases (hyaluronate lyases) have been identified in *S. pyogenes* and related species such as *S. equi* (8). Notably, *S. pyogenes* strains SF370.1 and 10,403 harbor the phage-encoded hyaluronate lyases HylP1 and HylP2, respectively, which have been implicated in tissue invasion and bacterial dissemination (9, 10). Hyaluronate lyases such as HylP2 facilitate bacterial invasion by degrading components of the host extracellular matrix. These enzymes primarily target hyaluronic acid (HA) and chondroitin sulfates, while exhibiting limited activity toward dermatan sulfates (11). Degradation of these glycosaminoglycans compromises host connective tissue integrity, facilitating bacterial spread and enhancing the dissemination of other phage-encoded toxins. The resulting breakdown products may also serve as nutrient sources that support bacterial proliferation at the sites of infection (12, 13). HylP2 has been biochemically characterized, and ascorbic acid has been identified as an inhibitor of its enzymatic activity (11). Additionally, phage-encoded hyaluronate lyases interact with the human transmembrane glycoprotein CD44, further contributing to host–pathogen interactions (12, 13).

.Medical management of *S. pyogenes* infections primarily relies on antibiotic therapy; however, the emergence of strains with reduced penicillin sensitivity underscores the urgent need for alternative strategies (14–16). Conventional antibiotics exert high selective pressure, driving resistance (17). In contrast, targeting HylP2 represents an anti-virulence strategy. By inhibiting the degradation of the host extracellular matrix, these agents may restrict bacterial dissemination and “disarm” the pathogen without exerting the bactericidal pressure that leads to resistance (18, 19). Consequently, HylP2 is a highly attractive molecular target for drug discovery.

Although efforts are underway to combat *S. pyogenes* infections using anti-virulence strategies, challenges remain in identifying effective and selective therapeutic agents. Numerous bioactive substances from diverse sources have been investigated for the treatment of infectious diseases. Microbial metabolites provide a valuable starting point for drug development because microorganisms can produce various natural compounds with considerable health benefits; these compounds currently constitute approximately half of all approved therapeutic agents (20). Natural products derived from myxobacteria, cyanobacteria, and fungi are the promising sources of anti-streptococcal agents owing to their extensive metabolic diversity and evolutionarily optimized chemical defense mechanisms. Myxobacteria have yielded hundreds of structurally diverse secondary metabolites, many with antibacterial activities, making them powerful reservoirs for novel antibiotics (21, 22). Cyanobacterial compounds and fungal secondary metabolites also exhibit broad-spectrum antimicrobial activities (23–25). Thus, these microorganisms represent valuable, underexplored resources for discovering compounds active against streptococci.

To identify potential inhibitors from these diverse natural sources, structure-based drug discovery has emerged as a critical approach. *In silico* workflows allow for the rapid characterization of drug–target complexes, providing mechanistic insights into ligand-binding behavior (26, 27). In this study, we employ an integrated pipeline utilizing molecular docking to predict binding orientations, followed by ADMET filtering to prioritize compounds with favorable pharmacokinetic profiles (28, 29). To overcome the limitations of static docking, molecular dynamics (MD) simulations are further employed to evaluate the stability and conformational flexibility of the complexes under physiological conditions (30). This integrated approach enhances the likelihood of identifying safe, stable, and biologically active lead compounds against *S. pyogenes*.

## 2 Materials and methods

### 2.1 Retrieval of hyaluronate lyase and preparation for molecular docking

The three-dimensional (3D) structure of hyaluronate lyase (HylP2; PDB: 2DP5, resolution: 3.55 Å) was obtained from the RCSB protein data bank^1^ in PDB format (31). This enzyme forms a single chain of 332 amino acids, with Gln261, Tyr264, and Arg279 as the active site residues. The enzyme was visualized using UCSF Chimera version 1.16 after removal of crystallographic water molecules (32). Thereafter, it was imported into the PyRx virtual screening tool,^2^ saved as an AutoDock macromolecule, and subsequently converted to PDBQT format (33, 34).

### 2.2 Collection of ligand 3D structures and preparation for molecular docking

In total, 118 compounds were retrieved from PubChem^3^ in SDF format (35). The molecules were selected based on documented antimicrobial or anti-streptococcal activities and their biosynthetic origins, which are known sources of bioactive secondary metabolites. The dataset included 27 compounds from bacteria and cyanobacteria, 36 from myxobacteria, 39 from fungi, and 13 established antibacterial agents (21, 36, 37). These organisms were targeted because they are well-recognized producers of structurally diverse natural products with reported activities against Gram-positive pathogens, including *Streptococcus* spp. ascorbic acid, triton X-100, and sodium dodecyl sulfate, which are the known inhibitors of hyaluronate lyase (11, 38), were included as controls to validate the docking protocol.

All compounds were imported into the PyRx virtual screening tool (See footnote 2) after successful download. The molecular structures were energy-minimized using the Universal Force Field (UFF) and subsequently converted to the PDBQT format for docking. AutoDock Vina settings were configured to ensure reproducibility. The exhaustiveness parameter was set to 8 to provide a balanced trade-off between computational cost and comprehensive exploration of the binding site (33, 34).

### 2.3 Molecular docking, interaction analysis, and visualization

Molecular docking was performed using the Vina wizard when the enzyme and ligands were successfully imported into the PyRx virtual screening tool (33). The grid dimensions were set in the Vina search space (Center X: 27.5716, Y: 18.1412, Z: 31.4922; Dimensions [Å] X: 33.5308, Y: 23.1604, Z: 23.5902) to contain the active site. This configuration allowed ligands to interact freely with the active-site residues to adopt a favorable binding conformation.

Redocking validation was performed using ascorbic acid, a co-crystallized inhibitor, to assess protocol reliability. The co-crystal ligand was extracted from the protein structure, energy-minimized using the UFF, and converted to the PDBQT format in PyRx. Docking was performed in AutoDock Vina (via PyRx v0.8) with an exhaustiveness value of 8, using a grid box sized to fully encompass the catalytic cleft. The top-scoring redocked pose yielded a root-mean-square deviation (RMSD) of 1.897 Å, which is within the acceptable threshold (<2.0 Å) for successful pose reproduction, and was centered at X = 15.085187, Y = 16.508875, Z = 32.766250. This confirmed that the docking workflow accurately replicated the native binding mode and was suitable for subsequent virtual screening analyses. The binding energies (kcal/mol) generated from the interaction between the enzyme and the ligands were recorded in a Microsoft Excel spreadsheet. The Biovia Discovery Studio Visualizer^4^ was used to visualize interactions between the hyaluronate lyase and the ligands after docking.

### 2.4 Computational prediction of ADMET parameters

The compounds were further subjected to *in silico* ADMET evaluation using ADMETlab2.0.^5^ These compounds were individually uploaded into the database in either SDF or simplified molecular-input line-entry system format. Their pharmacokinetic properties and drug-likeness were subsequently evaluated in accordance with Lipinski’s Rule of Five. Physicochemical properties and toxicity parameters were also assessed.

### 2.5 System preparation and force field selection for MD simulation

MD simulations were performed for 100 ns to evaluate the structural stability and binding persistence of six lead candidates: violacein, bikaverin, chlorotonil A, kulkenon, phenoxan, and xiamycin.

The 3D structure of HylP2 (PDB: 2DP5) was obtained, and preparation steps were performed, including the elimination of non-critical water molecules, heteroatoms, and metals (39). To ensure structural correctness, missing loops were modeled, and the protein was protonated to represent a physiological pH of 7.0. The energy minimization of the ligand (violacein, bikaverin, chlorotonil A, kulkenon, phenoxan, and xiamycin) geometries was achieved using the UFF (40). Ligand topology files and partial charges were produced using allied force field builders such as the Automated Topology Builder. To ensure consistent force field treatment within the complex, Amber ff14SB or Amber ff19SB force fields were applied to the protein parts, whereas the General Amber Force Field was used for the ligands (41, 42).

### 2.6 Solvation, ionization, and equilibration

For each complex, the orthorhombic or triclinic simulation box centered on the protein–ligand complex had a minimum distance of 10 Å (1.0 nm) from the box wall to the protein to prevent self-interactions during periodic boundary conditions. The systems were solvated using a standard three-point water model (TIP3P or SPC) to simulate a biological aqueous environment. Counterions (Na^+^ or Cl^−^) supplemented with 0.15 M NaCl were added in the case of uneven systems (43, 44). The systems underwent energy minimization for 5,000–10,000 steps to remove steric clashes, either by the steepest descent or conjugate gradient methods. Equilibration was performed in two stages: the NVT ensemble (constant number of particles, volume, and temperature), which allowed the system to reach the desired temperature of 300 K or 310.15 K; and the NPT ensemble (constant number of particles, pressure, and temperature), which stabilized the system’s density and pressure at 1 bar (45).

### 2.7 Production of MD simulations and trajectory analysis

The final MD simulations were performed for 100 ns using the NPT ensemble with a time step of 2 fs (46). All bonds involving hydrogen atoms, were constrained using the SHAKE or LINCS algorithms (47). The particle mesh Ewald method was used to handle electrostatic interactions with a standard 10-Å cutoff for non-bonded interactions. The coordinates and energy data were sampled every 10–100 ps to generate a complete dataset for trajectory analysis. Stability and conformational behavior were quantified using RMSD and root-mean-square fluctuation (RMSF), respectively, to evaluate structural stability and residue flexibility. The radius of gyration (Rg) was calculated to evaluate protein compactness. The 3D free-energy landscapes (FELs) and per-residue energy decomposition were used to locate the lowest-energy conformations and identify residues with the highest contribution to binding free energy (48).

## 3 Results

**Figure 1:**
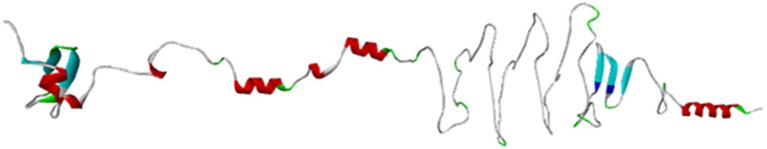
The 3D structure of bacteriophage-encoded hyaluronate lyase derived from *Streptococcus pyogenes* (viewed using Biovia Discovery Studio Visualizer).

Molecular docking of the compounds (ligands) against the crystal structure of *S. pyogenes*-derived hyaluronate lyase (Figure 1) was performed. The binding energies are presented in Tables 1–4.

**Table 1:** Binding affinity of compounds derived from bacteria and cyanobacteria against bacteriophage-encoded hyaluronate lyase from *Streptococcus pyogenes.*

Compounds	Binding affinity (kcal/mol)	Compounds	Binding energy (kcal/mol)
Coformycin	−4.9	Abietane	−6.1
Capolactin B	−4.8	Ambigol A	−5.5
Formycin	−4.9	Ambigol B	−5.4
Resveratrol	−5.5	Malyngolide	−4.3
Phenalamide A2	−5.7	Lyngbyoic acid	−4.2
Scutellarein	−5.9	pitinoic acid A	−3.9
Siastatin B	−4.9	Hapalindole	−6.4
Xiamycin	−7.3	anaephene B	−4.5
Violacein	−7.7	anaephene A	−4.2
Sarkomycin	−4.5	Anaephene C	−5
Anthranoside C	−6.0	Cylindrofridin A	−4
Apigenin	−6.0	Capolactin A	−4.5
Baicalein	−6.2	Triton-x100#	−4.7
Pinocembrin	−6.1	Ascorbic acid#	−3.6
Rosmarinic acid	−5.9	Sodium dodecyl sulfate#	−4.0

#Denotes reported inhibitors of hyaluronate lyase (11).

**Table 2:** Binding affinity of compounds from *Myxobacteria* against bacteriophage-encoded hyaluronate lyase from *S. pyogenes*.

Compounds	Binding affinity (kcal/mol)	Compounds	Binding energy (kcal/mol)
Epothilon D	−6.4	Ajudazol	−5
Kulkenon	−7.1	Althiomycin	−5
Myxochelin A	−4.7	Angiolactone	−5.5
Noricumazole C	−5.3	Aurachin E	−5.4
Phenoxan	−5.9	Carolacton	−5.3
Ratjadon	−5.2	Chlorotonil-A	−7.4
Thiangazole	−6.3	Corallorazine	−5
Sulfangolid C	−7.4	Cystobactamid	−6
Stipiamide	−4.7	Disciformycin A	−5.4
Soraphen F	−6.0	Enhygrolide B	−5.6
Labindole A	−4.4	Hyapyrone B	−6.7
Labindole B	−4.7	Hyalachelin	−4.8
3-chloro-9H-carbazole	−4.9	Hyaladione	−3.8
4-hydroxymethyl-quinoline	−6.4	Indiacen A	−5.2
Letermovir	−6.3	Indothiazinone	−4.8
Spirangien B	−6.0	Methyl indole-3-carboxylate	−4.4
Noricumazole A	−5.4	Melithiazol A	−4.4
Nannozinone B	−5.1	Nannozinone A	−5.5

**Table 3:** Binding affinity of fungi-derived compounds against bacteriophage-encoded hyaluronate lyase from *S. pyogenes*.

Compounds	Binding affinity (kcal/mol)	Compounds	Binding energy (kcal/mol)
Aureonitol	−4.0	Cordycepin	−4.5
Ganoderic acid	−5.2	Curvularin	−5.3
Velutin	−4.5	Citreorosein	−5.1
Deoxyfunicone	−5.5	Griseofulvin	−4.6
Fuscinarin	−5.8	Brefeldin A	−5
Hinnuliquinone	−6.5	Bikaverin	−5.8
Griseoxanthone C	−6.1	Xantocillin	−5.5
Kaempferol	−5.9	Aphidicolin	−5.2
10-methoxydihydrofuscin	−5.6	Cajanol	−4.7
Quercetin	−5.2	Lysergic acid	−5.3
Cytosporin A	−5.4	Ergothioneine	−3.9
Mellein	−5.2	Kojic acid	−3.5
6-Hydroxymellein	−4.5	Glutathione	−3.9
Sabinene	−3.9	Camptothecin	−6.2
cyclo(L-Phe-L-Pro)	−3.9	Tyrosol	−3.5
6-Pentyl-2H-pyran-2-one (6-Amyl-2-pyrone)	−3.9	Xanthone	−5.1
Emericellin	−5.6	2,4-Diacetylphloroglucinol	−4.2
cyclo(L-Pro-L-Val)	−4.1	Ergosterol	−6.8
Patulin	−3.7	Ergosterol peroxide	−6.3
Emodin	−5.3		

**Table 4:** Binding affinities of antibacterial compounds against bacteriophage-encoded hyaluronate lyase from *S. pyogenes*.

Compounds	Binding affinity (kcal/mol)
2-(2-chlorophenyl)-6-methoxy-4H-chromen-4-one	−5.9
1,3-Dicyclohexylurea	−4.5
(2Z)-2-(4-ethoxybenzylidene)-6-hydroxy-1-benzofuran-3(2H)-one	−4.9
6-[(3-methoxyphenyl)methoxy]-2-[(3,4,5-trimethoxyphenyl)methylidene]-1-benzofuran-3-one	−5.8
6-[2-(4-methoxyphenyl)-2-oxoethoxy]-2-[(2,4,5-trimethoxyphenyl)methylidene]-1-benzofuran-3-one	−5.5
7-hydroxy-3-(2-methoxyphenoxy)-2-methylchromen-4-one	−5.0
(2E)-3-(2,4-dimethoxyphenyl)-1-(2,5-dimethoxyphenyl)prop-2-en-1-one	−4.7
[2-[(2,3-Dimethoxyphenyl)methylidene]-3-oxo-1-benzofuran-6-yl] 2-methylpropanoate	−5.3
3-(2-methoxyphenyl)-4-oxo-4H-chromen-7-yl dimethylcarbamate	−5.7
[5-Hydroxy-3-(4-methoxyphenyl)-4-oxochromen-7-yl] 2,2-dimethylpropanoate	−5.9
5-hydroxy-4-oxo-2-phenyl-4H-chromen-7-yl dimethylcarbamate	−5.9
Methyl (1S,3S,4R)-3-hydroxy-4,7,7-trimethylbicyclo[2.2.1]heptane-1-carboxylate	−4.2
Methyl 2-{[(2Z)-2-[(3-methylphenyl)methylidene]-3-oxo-1-benzofuran-6-yl]oxy}acetate	−5.4

Binding energy is a function of the binding affinity of the ligand toward the enzyme. A lower value indicates a stronger affinity. The molecular docking of the compounds derived from bacteria and cyanobacteria against the *S. pyogenes*-derived hyaluronate lyase showed binding affinities ranging from −3.9 to −7.7 kcal/mol. The highest binding affinities were observed for violacein (−7.7 kcal/mol) and xiamycin (−7.3 kcal/mol). Baicalein, pinocembrin, apigenin, and anthranoside C showed binding affinities of −6.2, −6.1, and −6.0 kcal/mol, respectively. The positive controls (ascorbic acid, −3.6 kcal/mol; Triton X-100, −4.7 kcal/mol; sodium dodecyl sulfate, −4.0 kcal/mol) exhibited weaker binding, indicating that the predicted affinity of several natural compounds surpasses that of conventional inhibitors (Table 1). The compounds derived from myxobacteria revealed binding affinities ranging from −3.8 to −7.4 kcal/mol. Sulfangolid C, kulkenon, chlorotonil A, enhygrolide B, epothilon D, 4-hydroxymethyl-quinoline, and thiangazole showed binding affinities between −6.3 and −7.4 kcal/mol. Myxochelin A, noricumazole C, phenoxan, aurachin E, soraphen F, 3-chloro-9H-carbazole, melithiazol A, and carolacton showed binding affinities between −4.4 and −6.0 kcal/mol (Table 2). The binding affinities of fungi-derived compounds ranged from −3.5 to −6.8 kcal/mol. The highest binding affinities were observed for ergosterol (−6.8 kcal/mol), hinnuliquinone (−6.5 kcal/mol), ergosterol peroxide (−6.3 kcal/mol), and griseoxanthone C (−6.1 kcal/mol). In contrast, sabinene, cyclo(L-Phe-L-Pro), 6-Pentyl-2H-pyran-2-one, ergothioneine, and glutathione exhibited similar binding affinities of −3.9 kcal/mol, whereas brefeldin A, bikaverin, xantocillin, aphidicolin, lysergic acid, xanthone, kaempferol, 10-methoxydihydrofuscin, quercetin, and cytosporin A had binding affinities between −5.0 and −5.9 kcal/mol (Table 3). The binding affinities of the antibacterial compounds ranged from −4.2 to −5.9 kcal/mol. Within this group, the highest binding affinity (−5.9 kcal/mol) was observed for 5-hydroxy-4-oxo-2-phenyl-4H-chromen-7-yl dimethylcarbamate and 2-(2-chlorophenyl)-6-methoxy-4H-chromen-4-one (Table 4).

Visualization of compound–enzyme interactions revealed 0, 1, 2, 3, 4, 5, and 6 hydrogen bonds (H-bonds) in 26, 37, 24, 13, 8, 6, and 2 compounds, respectively. The most notable amino acid residues were Val185, Ser211, Met213, Leu215, Glu219, Thr224, Leu225, Lys226, Ile227, His229, Asn231, Asp239, Ala243, Ala244, Leu245, Leu247, Leu249, Gln261, Gly262, Ile263, Tyr264, Ile265, Leu275, Leu276, Arg279, Asn280, Leu281, Ser282, Phe286, Val288, Phe294, Ala296, Lys297, Glu298, Thr299, Ser300, and Gln301 (Supplementary Table S1). Among these, Gln261, Tyr264, and Arg279 constituted the active-site residues and were involved in interactions with kulkenon, noricumazole C, phenoxan, soraphen F, labindole B, aureonitol, velutin, ascorbic acid, kojic acid, emodin, citreorosein, griseofulvin, brefeldin A, bikaverin, xantocillin, lysergic acid, nannozinone B, anaephene B, xanthone, ajudazol, althiomycin, aurachin E, carolacton, cystobactamid, disciformycin A, and melithiazol A; comparable bond lengths were observed across these complexes. Violacein, xiamycin, sulfangolid C, and chlorotonil A formed between 0 and 4 H-bonds but did not interact directly with the active-site residues (Supplementary Table S1). The docking results indicated that the top-scoring ligands interacted with key catalytic residues, including Arg279 and Tyr264. These interactions suggest a possible active-site binding mode, which should be validated through enzyme kinetics studies.

**Figure 2:**
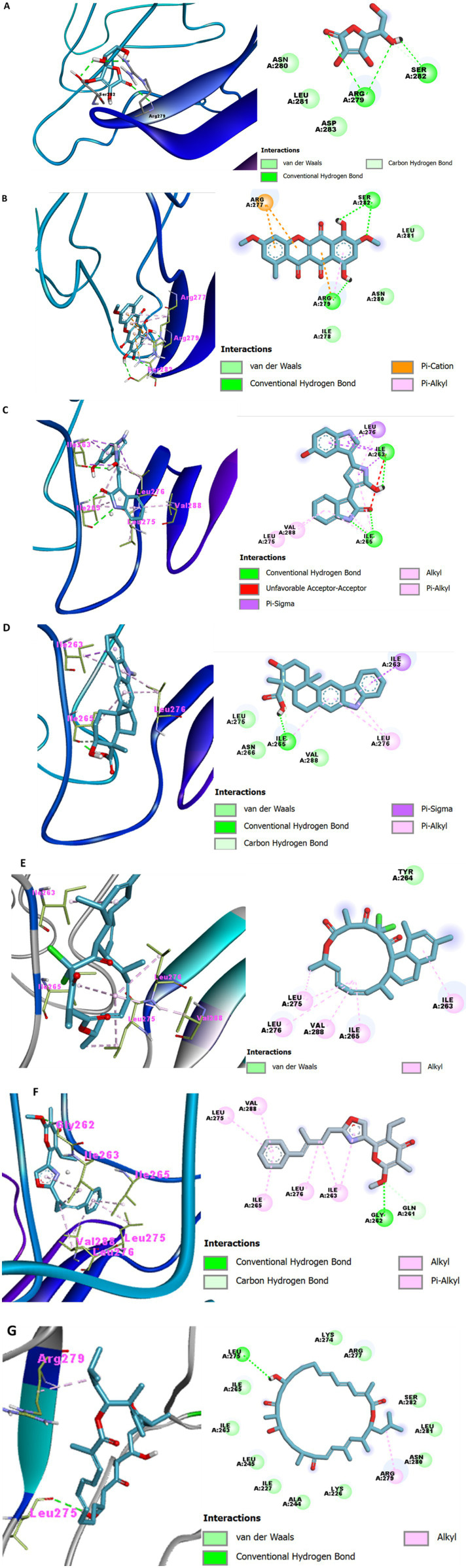
3-D (Left) and 2-D (Right) representations of the binding pose of ascorbic acid **(A)**, bikaverin **(B)**, violacein **(C)**, xiamycin **(D)**, chlorotinil **(E)**, phenoxan **(F)**, and kulkenon **(G)** in complex with bacteriophage-encoded hyaluronate lyase from *S. pyogenes*. 3-D (Left) and 2-D (Right) representations of the binding pose of ascorbic acid **(A)**, bikaverin **(B)**, violacein **(C)**, xiamycin **(D)**, chlorotinil **(E)**, phenoxan **(F)**, and kulkenon **(G)** in complex with bacteriophage-encoded hyaluronate lyase from *S. pyogenes*.

The detailed interactions of compounds forming at least two H-bonds with the active-site residues are presented in Figure 2. Noricumazole C formed six H-bonds with the enzyme, including conventional H-bonds with Thr224, Leu225, Ile227, and the active-site residue Arg279 (bond length: 2.16 Å). A similar pattern was observed for ascorbic acid, which formed six H-bonds—three of these bonds involved Arg279 (bond lengths: 2.02, 2.70, and 3.01 Å). Disciformycin A formed five H-bonds with Gln261, Gly262, and Tyr264, with bond lengths of 2.62, 2.49, and 2.10–2.98 Å, respectively. Similarly, brefeldin A formed two H-bonds with the active-site residues Gln261 (2.42 Å) and Tyr264 (2.06 Å). In addition to hydrogen bonding, these compounds exhibited hydrophobic and electrostatic interactions with the enzyme (Figure 2).

The *in silico* ADMET prediction analysis of the 26 compounds (including ascorbic acid) that interacted with at least one active-site residue is shown in Supplementary Table S2. Most compounds satisfied Lipinski’s rule and were not P-glycoprotein or CYP3A4 substrates. They were also predicted not to penetrate the blood–brain barrier. These compounds exhibited sufficient water solubility (−4.0–0.5 log mol/L) and lipophilicity (logD: 1–3), with moderate (5–10) clearance (Supplementary Table S2). Only eight compounds had suitable (0–3) n-octanol/water distribution coefficients. All compounds exhibited excellent (0.04–20 L/kg) volume distributions, with most having medium (0.3–0.7) to excellent (0–0.3) half-lives. Although a few compounds were predicted to cause eye irritation, many compounds were predicted to lack respiratory toxicity, human hepatotoxicity, or drug-induced liver injury and are non-carcinogenic. Only 10 compounds showed excellent (<90%) plasma protein-binding capacity.

**Figure 3:**
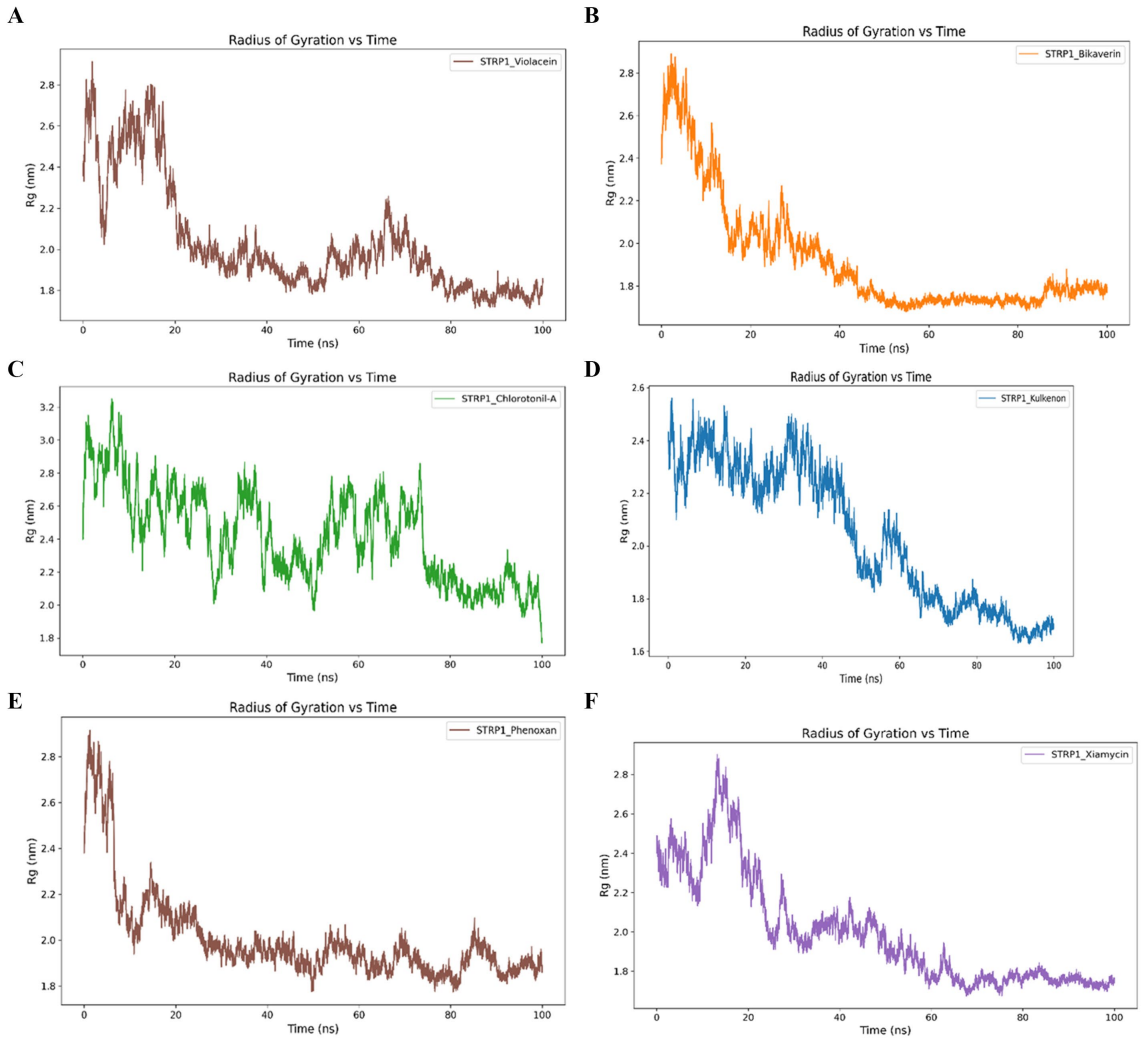
Radius of gyration (R_g_) profiles from molecular dynamics simulations of violacein **(A)**, bikaverin **(B)**, chlorotonil A **(C)**, kulkenon **(D)**, phenoxan **(E)**, and xiamycin **(F)** in complex with bacteriophage-encoded hyaluronate lyase from *S. pyogenes*. Radius of gyration (R_g_) profiles from molecular dynamics simulations of violacein **(A)**, bikaverin **(B)**, chlorotonil A **(C)**, kulkenon **(D)**, phenoxan **(E)**, and xiamycin **(F)** in complex with bacteriophage-encoded hyaluronate lyase from *S. pyogenes*.

MD simulations were conducted for 100 ns to gain insight into the dynamic stability and conformational behavior of the protein–ligand complexes. Rg analysis revealed initial expansion followed by structural compaction of the protein–ligand complexes during the simulation. The violacein- and bikaverin-bound complexes exhibited Rg peaks of approximately 2.8 nm, followed by a downward trend with noticeable fluctuations during the first 20 ns. Subsequently, they stabilized at approximately 1.8 nm and maintained this compact conformation throughout the 100-ns simulation. Chlorotonil A showed a high initial Rg peak of approximately 3.2 nm, which gradually decreased to approximately 1.8 nm over during the simulation, indicating progressive structural stabilization. Similarly, the kulkenon complex displayed an elevated Rg peak near 2.5 nm during the first 20 ns, before converging toward a stable, compact state. Phenoxan- and xiamycin-bound complexes followed comparable trends, beginning at around 2.4 nm and reaching transient peaks near 2.8 nm within the first 20 ns. These complexes subsequently maintained stable Rg values of approximately 1.8 nm for the remainder of the simulation period (Figure 3).

RMSD analysis revealed distinct equilibration and stability patterns among the protein–ligand complexes during the 100-ns simulation. The violacein-bound complex exhibited an RMSD peak of approximately 2.0 nm at around 20 ns, followed by pronounced fluctuations up to approximately 70 ns. Thereafter, the RMSD showed a downward trend and stabilized at approximately 1.5 nm toward the end of the simulation, indicating the attainment of a stable conformational state. The bikaverin complex displayed an early RMSD peak of approximately 1.5 nm within the initial phase of the simulation, followed by increased fluctuations and an upward peak exceeding 2.5 nm at approximately 20 ns. The complex then maintained a relatively stable RMSD signal around 2.5 nm throughout the remainder of the 100-ns period.

**Figure 4:**
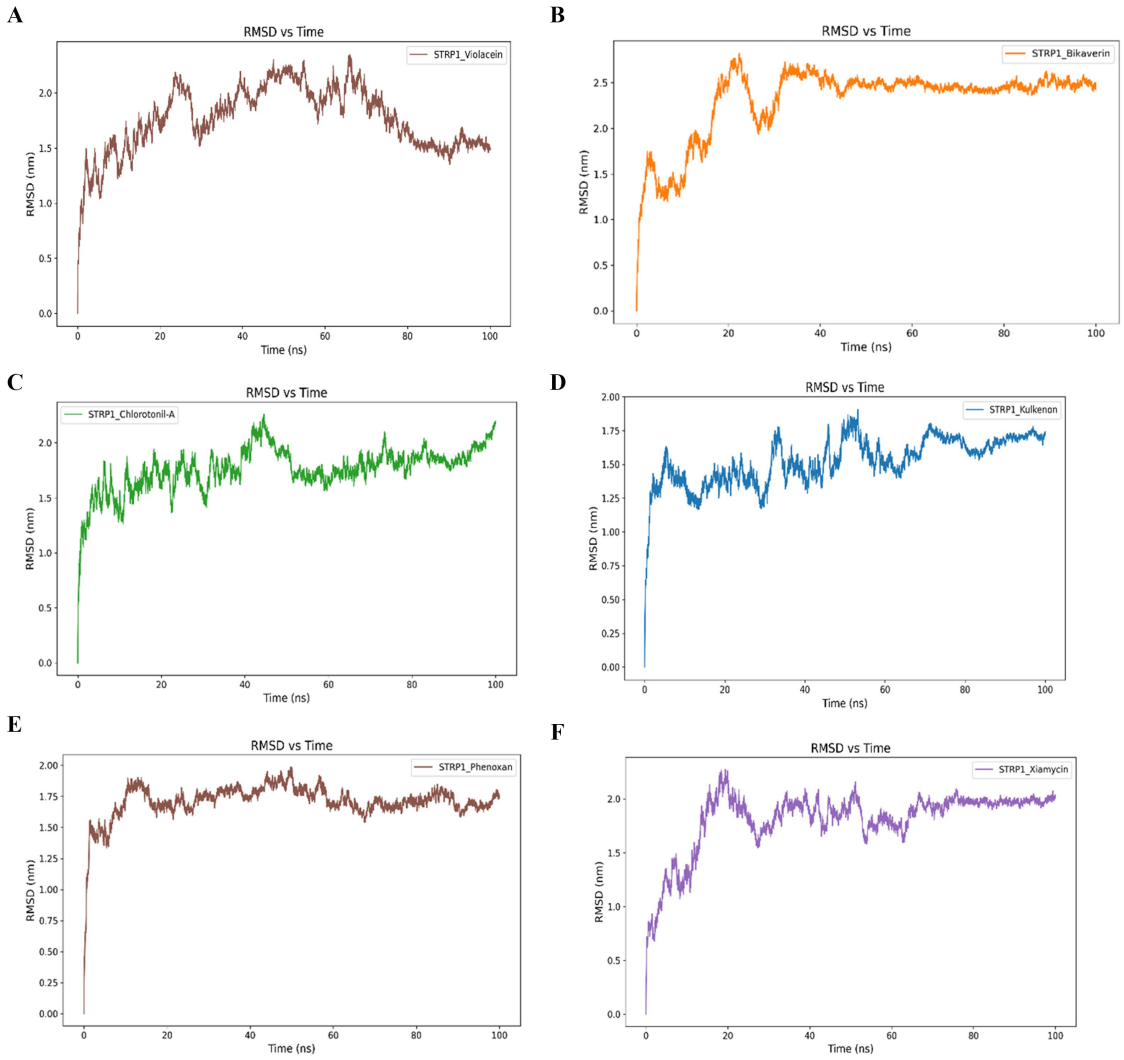
Root-mean-square deviation (RMSD) profiles obtained from the molecular dynamics simulations of violacein **(A)**, bikaverin **(B)**, chlorotonil A **(C)**, kulkenon **(D)**, phenoxan **(E)**, and xiamycin **(F)** in complex with bacteriophage-encoded hyaluronate lyase from *S. pyogenes.* Root-mean-square deviation (RMSD) profiles obtained from the molecular dynamics simulations of violacein **(A)**, bikaverin **(B)**, chlorotonil A **(C)**, kulkenon **(D)**, phenoxan **(E)**, and xiamycin **(F)** in complex with bacteriophage-encoded hyaluronate lyase from *S. pyogenes.*

A similar overall stabilization pattern was observed for chlorotonil A, kulkenon, phenoxan, and xiamycin complexes, which converged to stable RMSD values of approximately 1.7–1.8 nm over during the simulation. Specifically, the chlorotonil A complex exhibited a high initial RMSD peak at approximately 3.2 nm, followed by a gradual decrease to approximately 1.8 nm at the end of the simulation. Kulkenon showed a comparable trend, with a pronounced RMSD peak observed near 2.5 nm during the first 20 ns before stabilization. Phenoxan- and xiamycin-bound complexes followed similar trajectories, beginning at approximately 2.4 nm, reaching transient peaks near 2.8 nm within the first 20 ns, and subsequently stabilizing around 1.8 nm for the remainder of the simulation (Figure 4).

**Figure 5:**
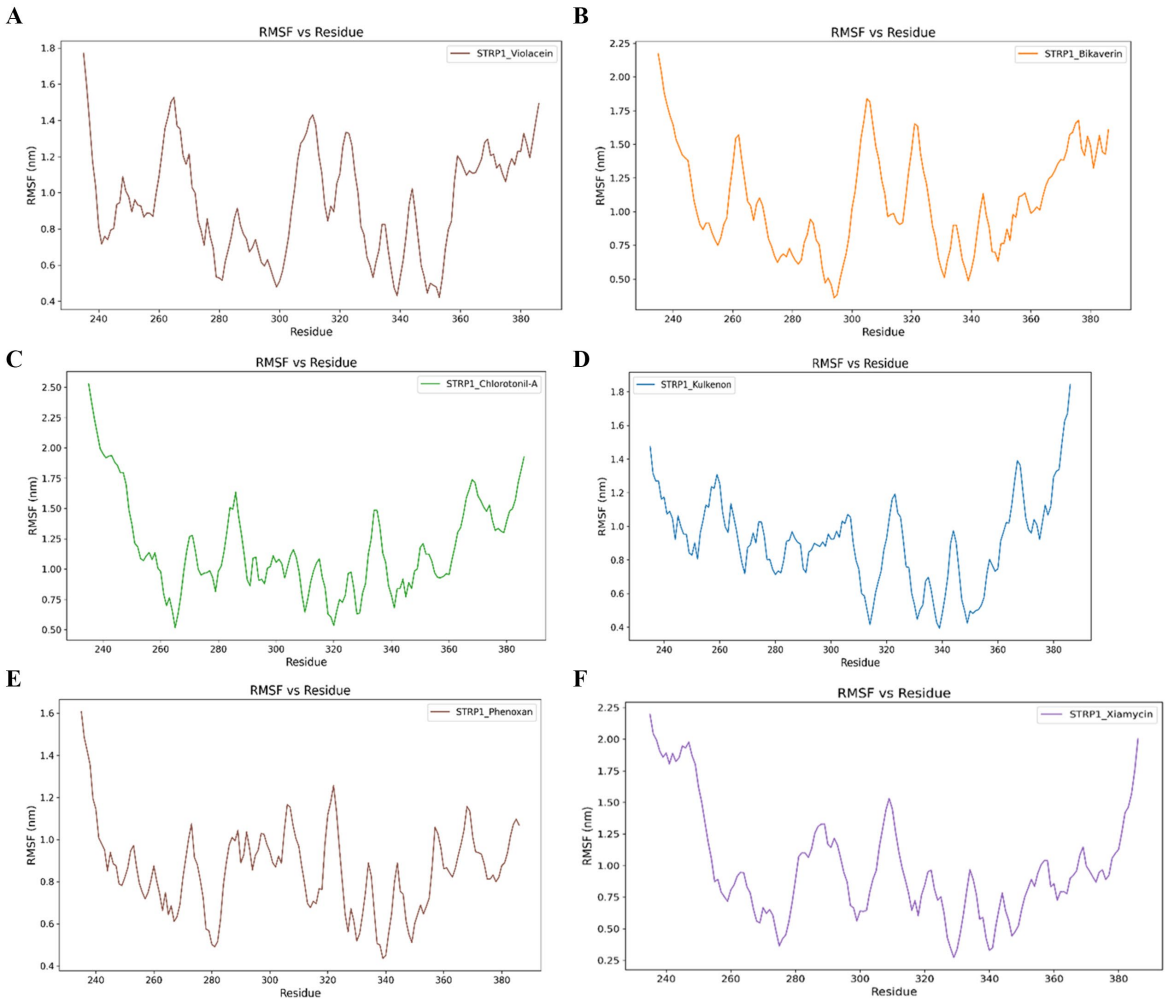
Root-mean-square fluctuation (RMSF) profiles obtained from MD simulations of violacein **(A)**, bikaverin **(B)**, chlorotonil-A **(C)**, kulkenon **(D)**, phenoxan **(E)**, and xiamycin **(F)** in complex with bacteriophage-encoded hyaluronate lyase derived from *S. pyogenes.*

RMSF analysis revealed that key residues of the protein, including those located within the catalytic region, exhibited consistently low fluctuations throughout the simulation period (Figure 5).

**Figure 6:**
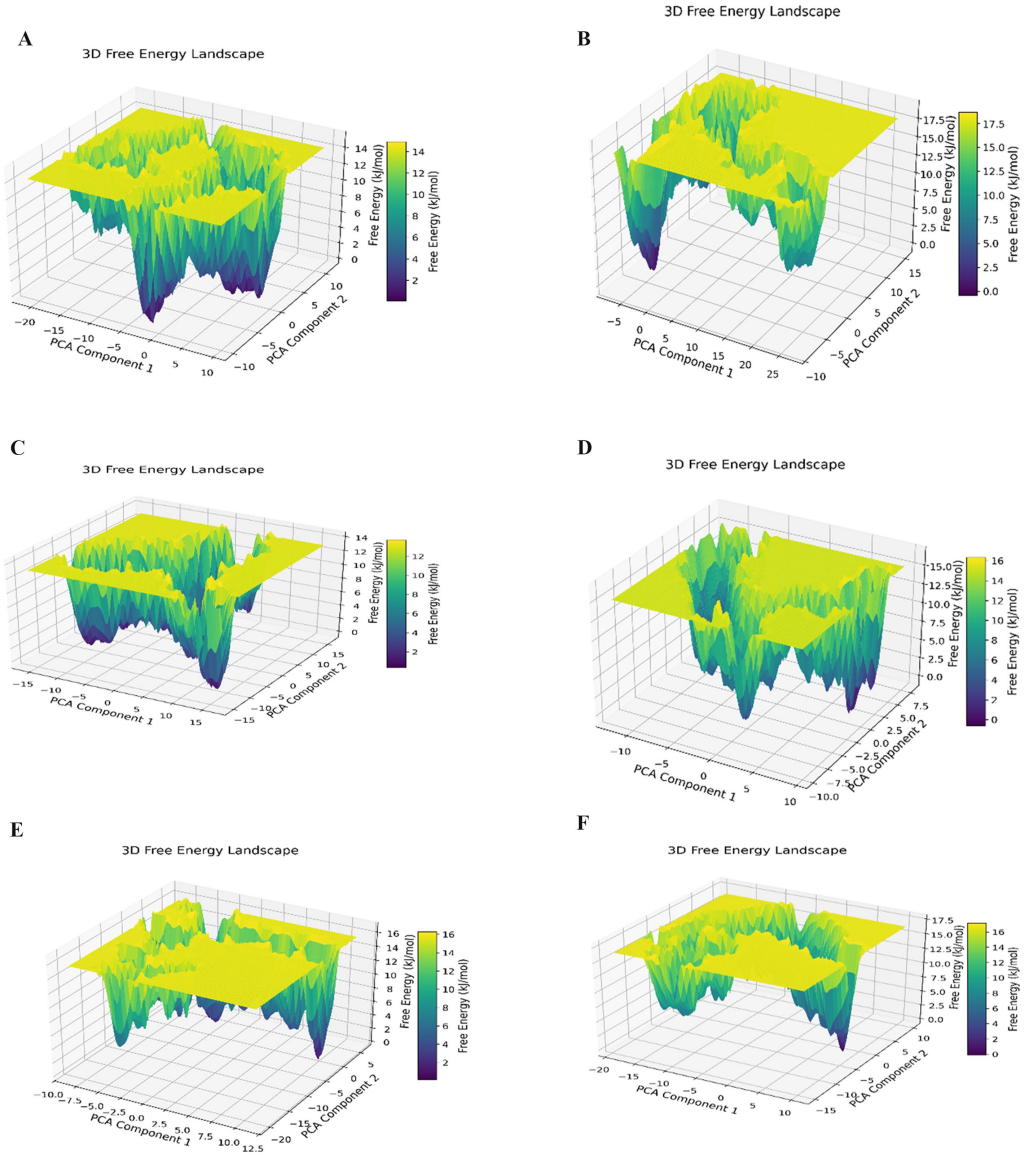
3D free-energy landscapes (FEL) profiles obtained from MD simulations of violacein **(A)**, bikaverin **(B)**, chlorotonil-A **(C)**, kulkenon **(D)**, phenoxan **(E)**, and xiamycin **(F)** in complex with bacteriophage-encoded hyaluronate lyase from *S. pyogenes*. 3D free-energy landscapes (FEL) profiles obtained from MD simulations of violacein **(A)**, bikaverin **(B)**, chlorotonil-A **(C)**, kulkenon **(D)**, phenoxan **(E)**, and xiamycin **(F)** in complex with bacteriophage-encoded hyaluronate lyase from *S. pyogenes*.

3D FEL analysis was used to evaluate the stability and dynamics of protein–ligand complexes during the simulation. FEL revealed well-defined low-energy basins corresponding to thermodynamically favorable stable conformational states. These minima represent the dominant conformations of the complexes, which exhibited limited conformational space and reduced flexibility owing to ligand binding. The lack of significant high-energy barriers suggested smooth transitions and overall stability during the simulation (Figure 6).

**Figure 7:**
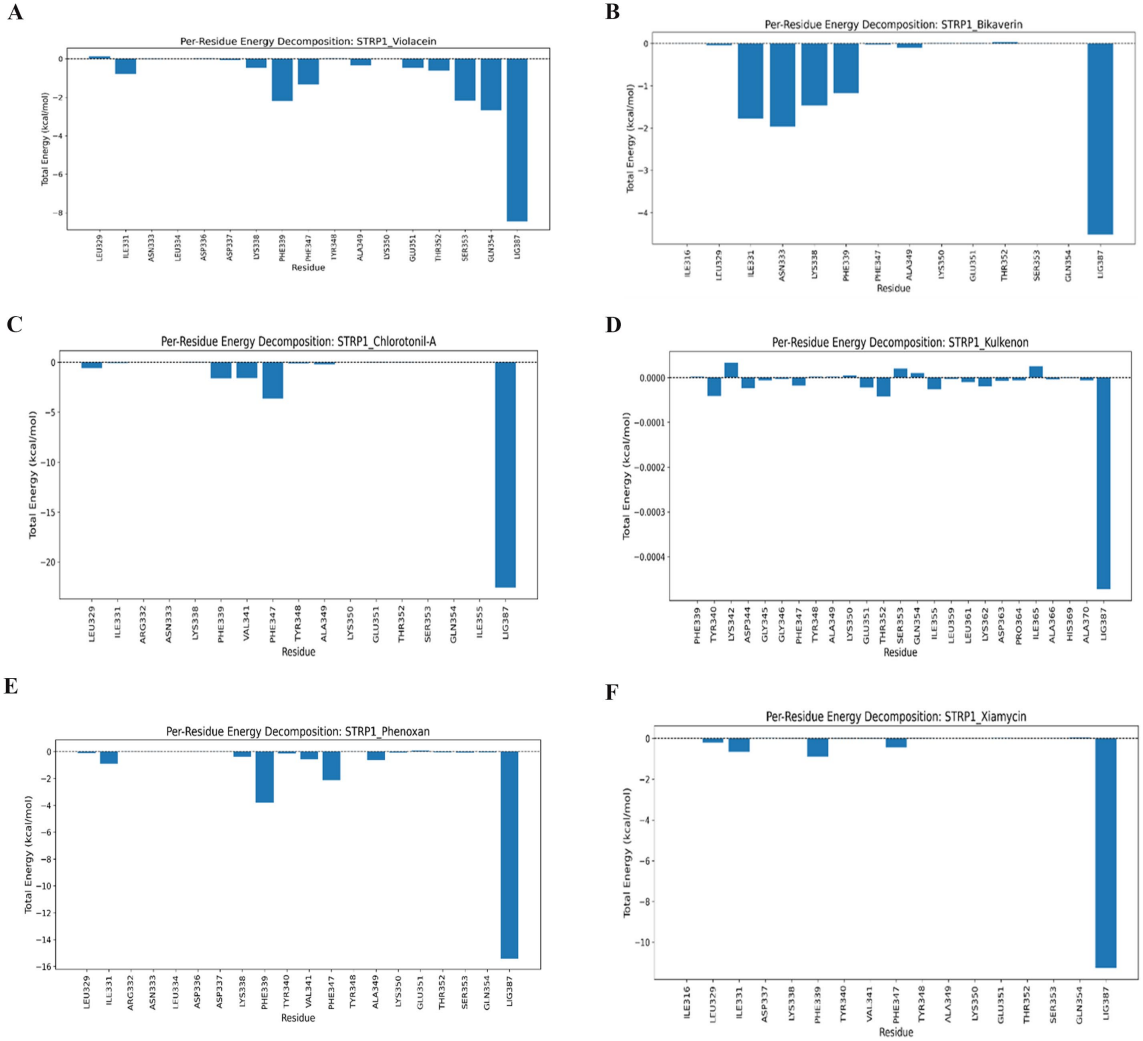
Per-residue energy decomposition profiles from MD simulations of violacein **(A)**, bikaverin **(B)**, chlorotonil A **(C)**, kulkenon **(D)**, phenoxan **(E)**, and xiamycin **(F)** in complex with bacteriophage-encoded hyaluronate lyase from *S. pyogenes*. Per-residue energy decomposition profiles from MD simulations of violacein **(A)**, bikaverin **(B)**, chlorotonil A **(C)**, kulkenon **(D)**, phenoxan **(E)**, and xiamycin **(F)** in complex with bacteriophage-encoded hyaluronate lyase from *S. pyogenes.*

Per-residue energy decomposition analysis was performed to identify key amino acid residues that contribute to ligand binding. Residues Phe339, Ser353, and Gln354 showed favorable binding contributions in the violacein complex, each exhibiting interaction energies of approximately −2.0 kcal/mol. The bikaverin complex displayed notable contributions from Ile331, Asn338, and Lys338, with interaction energies of approximately −2.0 kcal/mol, indicating stable residue–ligand interactions. The chlorotonil A complex showed a dominant contribution from Phe347, with a comparatively higher interaction energy of approximately −4.0 kcal/mol, suggesting strong residue involvement in ligand stabilization (Figure 7).

The kulkenon complex exhibited minimal residue-specific contributions, with interaction energies close to 0 kcal/mol, indicating weaker or more distributed interactions across the binding site. Phe339 was a major contributor to the phenoxan complex, with an interaction energy of approximately −4.0 kcal/mol, whereas Phe347 showed a moderate contribution (−2.0 kcal/mol). Similarly, residues Phe339, Ile331, and Phe347 contributed favorably to binding in the xiamycin-bound complex, each displaying interaction energies of approximately −1.0 kcal/mol. The ligand atom LIG387 consistently exhibited highly negative energy contributions across all complexes, highlighting its critical role in the stabilization of protein–ligand interactions (Figure 7).

## 4 Discussion

In this study, a few compounds had slightly lower binding affinities than previously reported inhibitors (ascorbic acid, Triton X-100, and sodium dodecyl sulfate). Most compounds interacted with *S. pyogenes*-derived hyaluronate lyase with higher binding affinities. The top-performing compounds (violacein, −7.7 kcal/mol; sulfangolid C, −7.4 kcal/mol; chlorotonil A, −7.4 kcal/mol; xiamycin, −7.3 kcal/mol; kulkenon, −7.1 kcal/mol) exhibited binding affinities broadly comparable to natural-product docking results reported in other antibacterial target studies. For instance, Chao et al. (49). evaluated natural compounds against three *Streptococcus*-associated targets (GlmU, PPAT, and RpoD). They reported binding affinities of −9.263 to −8.622 kcal/mol for GlmU, −8.038 to −7.25 kcal/mol for PPAT, and −7.154 to −6.521 kcal/mol for RpoD. The binding energies of the lead compounds identified in the present study align most closely with the affinity range reported for PPAT (−8.038 to −7.25 kcal/mol) and overlap with the upper range of affinities observed for RpoD (−7.154 to −6.521 kcal/mol). Although our compounds did not reach the extremely strong affinities obtained for GlmU (−9.263 to −8.622 kcal/mol), they demonstrated clinically relevant binding strengths, particularly considering that hyaluronate lyase is an anti-virulence rather than a bactericidal target.

Anti-virulence inhibitors are often reported to exhibit moderate binding energies. This ensures stable engagement with the catalytic pocket without exerting bactericidal pressure, which accelerates resistance development. Hassan et al. (50) identified a top hit of −7.2 kcal/mol [Sodium (1,5-dihydroxy-2-oxopyrrolidin-3-yl)-hydroxy-dioxidophosphanium] against *S. pneumoniae* alpha-enolase. Violacein, sulfangolid C, and chlorotonil A, the strongest compounds in this study, showed affinities that slightly exceeded or matched the highest affinities reported by Hassan et al. (50). This indicates that the natural molecules identified in this study interact with hyaluronate lyase at a binding strength comparable to that of validated in silico hits against other streptococcal targets.

Rehman et al. (51) have reported extremely high binding affinities for natural compounds docked to two essential *S. pyogenes* proteins. The interactions of DnaA–Sophorastilbene A and TCR protein–Aloin B had binding affinities of −21.31 kcal/mol and −18.02 kcal/mol, respectively. These strong binding scores are associated with essential replication and transcription proteins that possess highly conserved deep nucleotide-binding pockets. These proteins often display high docking scores owing to large hydrophobic cavities. In contrast, hyaluronate lyase is a secreted protein with a surface-exposed catalytic cleft designed for processing long glycosaminoglycan chains rather than for tight ligand encapsulation (52, 53). A similar pattern was observed by Rivera-Pérez et al. (54), who recorded markedly strong docking scores for inhibitors of the phosphotransferase system of *S. mutans*. Of these, ZINC15958489 and ZINC15968797 exhibited the highest binding affinities at −13.0 kcal/mol and −12.9 kcal/mol, respectively. These significantly stronger affinities correspond to molecules interacting with deeply recessed binding pockets in a membrane-associated transferase, which are considerably different from the topology of the hyaluronate lyase cleft.

Hydrogen bonding provides clear insights into the mechanisms whereby a molecule binds to an active site. Hydrogen bonding is instrumental in complex molecule inhibition because it ensures the stability of structures and functions (55). In this study, 26 compounds formed at least one H-bond with an active-site residue. Several top-ranking compounds interacted directly within the defined active-site pocket of hyaluronate lyase, including key catalytic and substrate-recognition residues (Gln261, Tyr264, and Arg279). Noricumazole C, disciformycin A, althiomycin, melithiazole A, griseofulvin, citreorosein, and bikaverin formed multiple H-bonds and hydrophobic contacts with these residues (particularly Arg279). These results suggest a stable and well-anchored binding mode. Their interaction patterns closely resembled those observed for ascorbic acid (11), supporting the relevance of their predicted orientations.

Engagement of active-site residues is especially important for hyaluronate lyase because inhibition requires obstruction of the catalytic groove responsible for glycosaminoglycan cleavage. Therefore, the ability of these compounds to interact with residues located within or immediately adjacent to the catalytic site provides a mechanistic rationale for their predicted inhibitory potential rather than relying solely on docking scores (55). In addition to hydrogen bonding, the stability and specificity of the ligand binding to hyaluronate lyase are also significantly affected by hydrophobic interactions, *π*–π stacking, and Van der Waals forces. Many of the top compounds, including violacein, xiamycin, sulfangolid C, chlorotonil A, and kulkenon, possess aromatic and nonpolar moieties that interact with the hydrophobic pockets within the catalytic cleft (11, 55). Aromatic rings in violacein and xiamycin facilitate π–π stacking interactions with aromatic residues, further stabilizing the ligand orientation and contributing to proper alignment with the catalytic residues Arg279 and Tyr264 (9, 12, 13).

Van der Waals forces fine-tune the ligand–enzyme complex by enabling close-range, weakly attractive interactions that complement H-bonds and hydrophobic contacts (56). These non-covalent interactions act synergistically to secure the ligand within the substrate-binding cleft and potentially enhance competitive inhibition by mimicking the interactions of natural substrates (11). Therefore, evaluating both H-bonds and these additional interactions elucidates the molecular determinants underlying the efficacy of hyaluronate lyase inhibitors. The predicted interactions of the top hits with the catalytic residues Arg279 and Tyr264 suggested that these compounds occupy the active site and potentially interfere with substrate binding. As molecular docking remains a predictive approach, biochemical assays such as enzyme inhibition kinetics are required to validate the inhibitory mechanisms and binding modes of the identified compounds.

To evaluate the biological activity of these compounds and predict whether they have favorable or harmful effects when utilized in pharmaceutical applications, ADMETLab2.0 was used to assess drug-like properties. Most of the compounds that engaged in H-bond interactions with at least one of the active-site residues passed Lipinski’s Rule of Five. Lipinski’s Rule of Five, which was used to determine whether the active compounds were orally active (57). The compounds were predicted to have good solubility, although only 10 had a plasma protein-binding capacity <90%, which may affect bioavailability. Early evaluation of drug solubility is crucial for drug development because low solubility hinders effective and complete oral absorption (58). Plasma protein-binding capacity can directly impact oral bioavailability because the free concentration of the drug is at risk when it binds to serum proteins (59). Overall, the compounds possessed good toxicological profiles, as they did not induce respiratory toxicity, human hepatotoxicity, or drug-induced liver injury, and were predicted to be non-carcinogenic.

Drug-induced liver damage has emerged as the most prevalent safety issue associated with drug withdrawal over the past 50 years and remains a primary reason medication are removed from the market to ensure patient safety (60). Minimal or no blood–brain barrier penetration may be necessary for medications with peripheral targets to prevent adverse effects on the central nervous system (61). The late and expensive discontinuation of drug development initiatives is frequently caused by adverse liver consequences in clinical trials (62, 63).

A smaller subset of compounds emerged as the most promising leads when the molecular docking results were integrated with ADMET and drug-likeness assessments. Violacein, xiamycin, kulkenon, chlorotonil A, and bikaverin demonstrated high binding affinities and favorable pharmacokinetic properties, including full compliance with Lipinski’s rule. Therefore, these molecules represent the lead candidates, combining strong predicted target engagement with acceptable drug-likeness and safety profiles. Phenoxan also exhibited good ADMET characteristics, although its binding energy was slightly lower than that of the top-performing compounds.

MD simulations added a temporal dimension to the docking results and permitted assessment of complex stability under simulated physiological conditions. The reliability of violacein, bikaverin, chlorotonil A, kulkenon, phenoxan, and xiamycin was dynamically validated by monitoring their interactions with the protein–ligand complex during a 100-ns MD trajectory.

The RMSD plots typically showed initial variation followed by convergence. This stabilization indicated that the ligands were firmly attached to the catalytic cleft and did not dissociate. Violacein and xiamycin showed synchronized trajectories after equilibration, indicating that their predicted binding orientations were reliable inhibitory poses. Rg plots for violacein and kulkenon showed a downward trend followed by a plateau, indicating that the protein adopted a denser form upon ligand binding. This structural compaction may isolate active-site residues from the surroundings, hindering natural substrate access to the production machinery. Local flexibility analysis via RMSF showed that important residues in the catalytic region exhibited low fluctuations throughout the simulation. The decrease in flexibility indicates that the presence of these natural compounds effectively “locks” the catalytic region, possibly preventing the conformational changes necessary for enzymatic activity.

The 3D FEL provided a strong thermodynamic argument for these interactions. The complexes were located at considerably deep and distinct energy minima. Per-residue energy decomposition indicated that the ligand molecules (LIG387) were primarily responsible for the largest negative total energy contribution to system stabilization. Residues such as Phe339 and Phe347 are pivotal anchors at the energy level of several complexes.

MD simulations identified violacein, xiamycin, and kulkenon as the most favorable compounds because of their highly stable and consistent convergence throughout the 100-ns trajectory. Rg profiling substantiated the maintenance of the compact structural state of the protein. FEL confirmed thermodynamic favorability; These compounds had markedly deep energy minima, further supported by their significant negative total energy contributions. These results indicate that these compounds were the most promising candidates for further experimental validation.

This study had some limitations: First, it was based entirely on *in silico* computational approaches. Therefore, the findings should be interpreted as predictive rather than conclusive. Molecular docking provides a theoretical estimate of ligand–protein binding affinity, but these scores do not directly correspond to actual enzymatic inhibition. Docking also assumes a largely rigid protein structure and may not fully capture the conformational flexibility of hyaluronate lyase or the dynamic nature of the enzymes long surface-exposed catalytic cleft. This was complemented by MD simulations. Additionally, ADMET predictions are based on quantitative structure–activity relationship models that carry inherent uncertainty and may not fully represent the actual pharmacokinetic behavior in biological systems. Consequently, the identified lead compounds require experimental validation using biochemical assays, enzyme inhibition studies, and cytotoxicity profiling to confirm their biological activity, potency, and safety. Further *in vitro* and *in vivo* studies are required before these candidates can be considered as viable therapeutic inhibitors.

## 5 Conclusion

The increasing prevalence of antibiotic resistance poses a major global health challenge, highlighting the need for alternatives to conventional bactericidal agents. Targeting virulence factors, such as the hyaluronate lyase derived from *S. pyogenes*, offers a promising anti-virulence approach that may reduce pathogenicity without exerting selective pressure for resistance. In this study, structure-based molecular docking, ADMET analysis, and 100-ns MD simulations were employed to evaluate the binding potential of 118 natural compounds.

Virtual screening identified violacein, chlorotonil A, sulfangolid C, xiamycin, and kulkenon as high-affinity candidates with favorable ADMET properties. Mechanistic analysis revealed that these top hits consistently interacted with the key catalytic residues Arg279 and Tyr264. MD simulations further validated these findings. Violacein, xiamycin, and kulkenon demonstrated exceptional structural stability and convergent trajectories, effectively “locking” the catalytic region in a compact non-functional state. The direct engagement with the catalytic pocket and stable energetic minima identified through the FEL suggest that these compounds act as competitive inhibitors, thereby preventing the enzyme from processing host glycosaminoglycans. This mechanism aligns with those of anti-virulence therapy strategies and has the potential to reduce host tissue degradation and bacterial dissemination without imposing selective pressure on the bacteria.

Although these findings offer a robust foundation for the development of hyaluronate lyase-targeted inhibitors, they remain predictive and are derived from computational methods. Therefore, *in vitro* enzyme assays, cytotoxicity screening, and *in vivo* infection models are required to validate these molecules as true inhibitors and to establish their therapeutic potential. Overall, this study identified promising natural product scaffolds and provided dynamic mechanistic insights into their modes of action, supporting the continued exploration of anti-virulence strategies against *S. pyogenes.*

## Data Availability

The original contributions presented in this study are included in this article/Supplementary material, further inquiries can be directed to the corresponding author.

## Footnotes

1
https://www.rcsb.org/

2 version 0.8; https://pyrx.sourceforge.io

3
https://pubchem.ncbi.nlm.nih.gov/

4
https://discover.3ds.com/discovery-studio-visualizer-download

5
https://admetmesh.scbdd.com/

## References

[1] Ibrahim J Eisen JA Jospin G Coil DA Khazen G Tokajian S. Genome analysis of *Streptococcus pyogenes* associated with pharyngitis and skin infections. PLoS One. (2016) 11:e0168177. doi: 10.1371/journal.pone.0168177, 27977735 PMC5158041

[2] Kanwal S Vaitla P. Streptococcus pyogenes. Treasure Island, FL: StatPearls Publishing (2025). 32119415

[3] Brouwer S Rivera-Hernandez T Curren BF Harbison-Price N De Oliveira DMP Jespersen MG . Pathogenesis, epidemiology and control of group a Streptococcus infection. Nat Rev Microbiol. (2023) 21:431–47. doi: 10.1038/s41579-023-00865-7, 36894668 PMC9998027

[4] Shuwen H Kefeng D. Intestinal phages interact with bacteria and are involved in human diseases. Gut Microbes. (2022) 14:2113717. doi: 10.1080/19490976.2022.2113717, 36037202 PMC9427043

[5] Niemann H Birch-Andersen A Kjems E Mansa B Stirm S. Streptococcal bacteriophage 12/12-borne hyaluronidase and its characterization as a lyase (EC 4.2.99.1) by means of streptococcal hyaluronic acid and purified bacteriophage suspensions. Acta Pathol Microbiol Scand: B, Microbiol. (1976) 84:145–53. doi: 10.1111/j.1699-0463.1976.tb01917.x, 793293

[6] Canchaya C Desiere F McShan WM Ferretti JJ Parkhill J Brüssow H. Genome analysis of an inducible prophage and prophage remnants integrated in the *Streptococcus pyogenes* strain SF370. Virology. (2002) 302:245–58. doi: 10.1006/viro.2002.1570, 12441069

[7] Hynes WL Walton SL. Hyaluronidases of gram-positive bacteria. FEMS Microbiol Lett. (2000) 183:201–7. doi: 10.1111/j.1574-6968.2000.tb08958.x, 10675584

[8] Kreil G. Hyaluronidases—a group of neglected enzymes. Protein Sci. (1995) 4:1666–9. doi: 10.1002/pro.5560040902, 8528065 PMC2143229

[9] Hynes WL Hancock L Ferretti JJ. Analysis of a second bacteriophage hyaluronidase gene from *Streptococcus pyogenes*: evidence for a third hyaluronidase involved in extracellular enzymatic activity. Infect Immun. (1995) 63:3015–20. doi: 10.1128/iai.63.8.3015-3020.1995, 7622224 PMC173410

[10] Smith NL Taylor EJ Lindsay AM Charnock SJ Turkenburg JP Dodson EJ . Structure of a group a streptococcal phage-encoded virulence factor reveals a catalytically active triple-stranded beta-helix. Proc Natl Acad Sci USA. (2005) 102:17652–7. doi: 10.1073/pnas.0504782102, 16314578 PMC1308890

[11] Mishra P Prem Kumar R Ethayathulla AS Singh N Sharma S Perbandt M . Polysaccharide binding sites in hyaluronate lyase–crystal structures of native phage-encoded hyaluronate lyase and its complexes with ascorbic acid and lactose. FEBS J. (2009) 276:3392–402. doi: 10.1111/j.1742-4658.2009.07065.x, 19438710

[12] Singh SK Bharati AP Singh N Pandey P Joshi P Singh K . The prophage-encoded hyaluronate lyase has broad substrate specificity and is regulated by the N-terminal domain. J Biol Chem. (2014) 289:35225–36. doi: 10.1074/jbc.M113.507673, 25378402 PMC4271211

[13] Singh SK Malhotra S Akhtar MS. Characterization of hyaluronic acid specific hyaluronate lyase (HylP) from *Streptococcus pyogenes*. Biochimie. (2014) 102:203–10. doi: 10.1016/j.biochi.2014.03.012, 24721581

[14] Chochua S Metcalf B Li Z Mathis S Tran T Rivers J . Invasive group a streptococcal penicillin binding protein 2× variants associated with reduced susceptibility to β-lactam antibiotics in the United States, 2015-2021. Antimicrob Agents Chemother. (2022) 66:e0080222. doi: 10.1128/aac.00802-22, 35969070 PMC9487518

[15] Hayes A Lacey JA Morris JM Tong SYC. Restricted sequence variation in *Streptococcus pyogenes* penicillin binding proteins. mSphere. (2020) 5:e00090. doi: 10.1128/mSphere.00090-20, 32350098 PMC7193039

[16] Vannice KS Ricaldi J Nanduri S Fang FC Lynch JB Bryson-Cahn C . *Streptococcus pyogenes* pbp2x mutation confers reduced susceptibility to β-lactam antibiotics. Clin Infect Dis. (2020) 71:201–4. doi: 10.1093/cid/ciz1000, 31630171 PMC7167332

[17] Ventola CL. The antibiotic resistance crisis: part 1: causes and threats. PT. (2015) 40:277–83. 25859123 PMC4378521

[18] Clatworthy AE Pierson E Hung DT. Targeting virulence: a new paradigm for antimicrobial therapy. Nat Chem Biol. (2007) 3:541–58. doi: 10.1038/nchembio.2007.24, 17710100

[19] Rasko DA Sperandio V. Anti-virulence strategies to combat bacteria-mediated disease. Nat Rev Drug Discov. (2010) 9:117–28. doi: 10.1038/nrd3013, 20081869

[20] Ramírez-Rendon D Passari AK Ruiz-Villafán B Rodríguez-Sanoja R Sánchez S Demain AL. Impact of novel microbial secondary metabolites on the pharma industry. Appl Microbiol Biotechnol. (2022) 106:1855–78. doi: 10.1007/s00253-022-11821-5, 35188588 PMC8860141

[21] Mulwa LS Stadler M. Antiviral compounds from Myxobacteria. Microorganisms. (2018) 6:73. doi: 10.3390/microorganisms6030073, 30029487 PMC6163824

[22] Bhat MA Mishra AK Bhat MA Banday MI Bashir O Rather IA . Myxobacteria as a source of new bioactive compounds: a perspective study. Pharmaceutics. (2021) 13:1265. doi: 10.3390/pharmaceutics13081265, 34452226 PMC8401837

[23] Swain SS Paidesetty SK Padhy RN. Antibacterial, antifungal and antimycobacterial compounds from cyanobacteria. Biomed Pharmacother. (2017) 90:760–76. doi: 10.1016/j.biopha.2017.04.030, 28419973

[24] Cock IE Cheesman MJ. A review of the antimicrobial properties of cyanobacterial natural products. Molecules. (2023) 28:7127. doi: 10.3390/molecules28207127, 37894609 PMC10608859

[25] Grabowski Ł Wiśniewska K Żabińska M Konarzewska M Zielenkiewicz M Rintz E . Cyanobacteria and their metabolites - can they be helpful in the fight against pathogenic microbes? Blue Biotechnol. (2024) 1:4. doi: 10.1186/s44315-024-00003-9

[26] Agu PC Afiukwa CA Orji OU Ezeh EM Ofoke IH Ogbu CO . Molecular docking as a tool for the discovery of molecular targets of nutraceuticals in diseases management. Sci Rep. (2023) 13:13398. doi: 10.1038/s41598-023-40160-2, 37592012 PMC10435576

[27] Meng XY Zhang HX Mezei M Cui M. Molecular docking: a powerful approach for structure-based drug discovery. Curr Comput Aided Drug Des. (2011) 7:146–57. doi: 10.2174/157340911795677602, 21534921 PMC3151162

[28] Nivatya HK Singh A Kumar N Sonam Sharma L Singh V . Assessing molecular docking tools: understanding drug discovery and design. Future J Pharm Sci. (2025) 11:111. doi: 10.1186/s43094-025-00862-y

[29] Millan-Casarrubias EJ García-Tejeda YV González-De la Rosa CH Ruiz-Mazón L Hernández-Rodríguez YM Cigarroa-Mayorga OE. Molecular docking and pharmacological in silico evaluation of camptothecin and related ligands as promising HER2-targeted therapies for breast cancer. Curr Issues Mol Biol. (2025) 47:193. doi: 10.3390/cimb47030193, 40136447 PMC11941075

[30] Hollingsworth SA Dror RO. Molecular dynamics simulation for all. Neuron. (2018) 99:1129–43. doi: 10.1016/j.neuron.2018.08.011, 30236283 PMC6209097

[31] Burley SK Berman HM Kleywegt GJ Markley JL Nakamura H Velankar S. Protein data Bank (PDB): the single global macromolecular structure archive In: Burley SK, editor. Protein Crystallography: Methods and Protocols: Methods in Molecular Biology, vol. 1607. New York, NY): Humana Press (2017). 627–41. 10.1007/978-1-4939-7000-1_26 PMC5823500 28573592

[32] Butt SS Badshah Y Shabbir M Rafiq M. Molecular docking using chimera and autodock vina software for nonbioinformaticians. JMIR Bioinform Biotechnol. (2020) 1:e14232. doi: 10.2196/14232, 38943236 PMC11168238

[33] Trott O Olson AJ. AutoDock Vina: improving the speed and accuracy of docking with a new scoring function, efficient optimization, and multithreading. J Comput Chem. (2010) 31:455–61. doi: 10.1002/jcc.21334, 19499576 PMC3041641

[34] Kondapuram SK Sarvagalla S Coumar MS. Docking-based virtual screening using PyRx tool: autophagy target Vps34 as a case study In: Coumar MS, editor. Molecular docking for computer-aided drug design. Cambridge, MA: Academic Press (2021). 463–77.

[35] Xie XQS. Exploiting PubChem for virtual screening. Expert Opin Drug Discov. (2010) 5:1205–20. doi: 10.1517/17460441.2010.524924, 21691435 PMC3117665

[36] Linnakoski R Reshamwala D Veteli P Cortina-Escribano M Vanhanen H Marjomäki V. Antiviral agents from fungi: diversity, mechanisms and potential applications. Front Microbiol. (2018) 9:2325. doi: 10.3389/fmicb.2018.02325, 30333807 PMC6176074

[37] Raihan T Rabbee MF Roy P Choudhury S Baek KH Azad AK. Microbial metabolites: the emerging hotspot of antiviral compounds as potential candidates to avert viral pandemic alike COVID-19. Front Mol Biosci. (2021) 8:732256. doi: 10.3389/fmolb.2021.732256, 34557521 PMC8452873

[38] El-Safory NS Lee GC Lee CK. Characterization of hyaluronate lyase from *Streptococcus pyogenes* bacteriophage H4489A. Carbohydr Polym. (2011) 84:1182–91. doi: 10.1016/j.carbpol.2011.01.019

[39] Roe DR Brooks BR. A protocol for preparing explicitly solvated systems for stable molecular dynamics simulations. J Chem Phys. (2020) 153:054123. doi: 10.1063/5.0013849, 32770927 PMC7413747

[40] Artemova S Jaillet L Redon S. Automatic molecular structure perception for the universal force field. J Comput Chem. (2016) 37:1191–205. doi: 10.1002/jcc.24309, 26927616

[41] Tian C Kasavajhala K Belfon KA Raguette L Huang H Migues AN . ff19SB: amino-acid-specific protein backbone parameters trained against quantum mechanics energy surfaces in solution. J Chem Theory Comput. (2019) 16:528–52. doi: 10.1021/acs.jctc.9b00591, 31714766 PMC13071887

[42] He X Man VH Yang W Lee TS Wang J. A fast and high-quality charge model for the next generation general AMBER force field. J Chem Phys. (2020) 153:114502. doi: 10.1063/5.0019056, 32962378 PMC7728379

[43] Rizzato S Gavezzotti A Lo Presti L. Molecular dynamics simulation of molecular crystals under anisotropic compression: bulk and directional effects in anthracene and paracetamol. Cryst Growth Des. (2020) 20:7421–8. doi: 10.1021/acs.cgd.0c01098

[44] Sengupta A Li Z Song LF Li P Merz KM Jr. Parameterization of monovalent ions for the OPC3, OPC, TIP3P-FB, and TIP4P-FB water models. J Chem Inf Model. (2021) 61:869–80. doi: 10.1021/acs.jcim.0c01390, 33538599 PMC8173365

[45] Ahmad S Ahmad F Abideen SA Khan K Irfan M Siddique F . Dynamics understanding of novel solvated drug molecules against emerging *Burkholderia cepacia* infections in immunocompromised patients. Results Chem. (2025) 15:102250. doi: 10.1016/j.rechem.2025.102250, 40503072 PMC12149026

[46] Krieger E Vriend G. New ways to boost molecular dynamics simulations. J Comput Chem. (2015) 36:996–1007. doi: 10.1002/jcc.23899, 25824339 PMC6680170

[47] Elber R Ruymgaart AP Hess B. SHAKE parallelization. Eur Phys J Spec Top. (2011) 200:211–23. doi: 10.1140/epjst/e2011-01525-9, 22368766 PMC3285512

[48] Alawi MM Alyazidi RM Bajrai LH Gattan HS Alandijany TA Al-Zahrani IA . Structural insights into natural compound inhibitors of the human metapneumovirus nucleocapsid protein via molecular dynamics and free energy landscape analyses. Sci Rep. (2025) 15:38654. doi: 10.1038/s41598-025-22428-x, 41188391 PMC12586523

[49] Chao P Zhang X Zhang L Yang A Wang Y Chen X. Integration of molecular docking and molecular dynamics simulations with subtractive proteomics approach to identify the novel drug targets and their inhibitors in *Streptococcus gallolyticus*. Sci Rep. (2024) 14:14755. doi: 10.1038/s41598-024-64769-z, 38926437 PMC11208513

[50] Hassan M Baig AA Attique SA Abbas S Khan F Zahid S . Molecular docking of alpha-enolase to elucidate the promising candidates against *Streptococcus pneumoniae* infection. DARU J Pharm Sci. (2021) 29:73–84. doi: 10.1007/s40199-020-00384-3, 33537864 PMC8149539

[51] Rehman A Wang X Ahmad S Shahid F Aslam S Ashfaq UA . In silico core proteomics and molecular docking approaches for the identification of novel inhibitors against *Streptococcus pyogenes*. Int J Environ Res Public Health. (2021) 18:11355. doi: 10.3390/ijerph182111355, 34769873 PMC8582943

[52] Li S Jedrzejas MJ. Hyaluronan binding and degradation by *Streptococcus agalactiae* hyaluronate lyase. J Biol Chem. (2001) 276:41407–16. doi: 10.1074/jbc.M106634200, 11527972

[53] Hynes W Sloan M. Secreted extracellular virulence factors In: Ferretti JJ Stevens DL Fischetti VA, editors. *Streptococcus pyogenes*: Basic biology to clinical manifestations. Oklahoma City, OK: University of Oklahoma Health Sciences Center (2016) 26866208

[54] Rivera-Pérez WA Yépes-Pérez AF Martínez-Pabón MC. Molecular docking and in silico studies of the physicochemical properties of potential inhibitors for the phosphotransferase system of *Streptococcus mutans*. Arch Oral Biol. (2019) 98:164–75. doi: 10.1016/j.archoralbio.2018.09.020, 30500666

[55] Ibrahim ZYU Uzairu A Shallangwa G Abechi S. Molecular docking studies, drug-likeness and *in-silico* ADMET prediction of some novel β-aminoalcohol grafted 1, 4, 5-trisubstituted 1, 2, 3-triazoles derivatives as elevators of p53 protein levels. Sci Afr. (2020) 10:e00570. doi: 10.1016/j.sciaf.2020.e00570

[56] Leach AR. Molecular modelling: Principles and applications. Harlow, UK: Pearson Education (2001).

[57] Lipinski CA Lombardo F Dominy BW Feeney PJ. Experimental and computational approaches to estimate solubility and permeability in drug discovery and development settings. Adv Drug Deliv Rev. (2001) 46:3–26. doi: 10.1016/s0169-409x(00)00129-0, 11259830

[58] Fink C Sun D Wagner K Schneider M Bauer H Dolgos H . Evaluating the role of solubility in oral absorption of poorly water-soluble drugs using physiologically-based pharmacokinetic modeling. Clin Pharmacol Ther. (2020) 107:650–61. doi: 10.1002/cpt.1672, 31608434 PMC7158207

[59] Charlier B Coglianese A De Rosa F De Grazia U Operto FF Coppola G . The effect of plasma protein binding on the therapeutic monitoring of antiseizure medications. Pharmaceutics. (2021) 13:1208. doi: 10.3390/pharmaceutics13081208, 34452168 PMC8401952

[60] Alempijevic T Zec S Milosavljevic T. Drug-induced liver injury: do we know everything? World J Hepatol. (2017) 9:491–502. doi: 10.4254/wjh.v9.i10.491, 28443154 PMC5387361

[61] Upadhyay RK. Drug delivery systems, CNS protection, and the blood brain barrier. Biomed Res Int. (2014) 2014:869269. doi: 10.1155/2014/869269, 25136634 PMC4127280

[62] Andrade RJ Robles M Fernández-Castañer A López-Ortega S López-Vega MC Lucena MI. Assessment of drug-induced hepatotoxicity in clinical practice: a challenge for gastroenterologists. World J Gastroenterol. (2007) 13:329–40. doi: 10.3748/wjg.v13.i3.329, 17230599 PMC4065885

[63] David S Hamilton JP. Drug-induced liver injury. US Gastroenterol Hepatol Rev. (2010) 6:73–80. 21874146 PMC3160634

