# Supplementary material for: In silico interaction analysis of selected natural compounds with bacteriophage-encoded hyaluronate lyase from Streptococcus pyogenes
Source: Front Med (Lausanne). 2026 Feb 16;13:1709023. doi: 10.3389/fmed.2026.1709023 (PMC12950598; doi:10.3389/fmed.2026.1709023)
Supplement: Supplementary file 1 [file Table_1.pdf]

Supplementary information

In silico interaction analysis of selected natural compounds with bacteriophage-encoded hyaluronate lyase from *Streptococcus pyogenes*

Samia S. Alkhalil *

Department of Medical Laboratory Sciences, College of Applied Medical Sciences, Shaqra University, Alquwayiyah, Riyadh, Saudi Arabia

*** Correspondence:**
Samia S. Alkhalil
salkhalil@su.edu.sa

KEYWORDS: bacteriophage, hyaluronate lyase, molecular docking, molecular dynamics, natural compounds, *Streptococcus pyogenes*

Supplementary Material

Table S1. Visualization of interactions between the compounds and bacteriophage-encoded hyaluronate lyase derived from *S. pyogenes*

Compound	Interacting AA residue(s)	AA residue(s) forming H-bonds	No. of H-bonds	Bond length (Å)
2-(2-chlorophenyl)-6-methoxy-4H-chromen-4-one	Ile263, Ile265, Leu275, Leu276, Val288	Ile265	1	2.0
1,3-Dicyclohexylurea			0	
(2Z)-2-(4-ethoxybenzylidene)-6-hydroxy-1-benzofuran-3(2H)-one	Ile265, Val288		0	
6-[(3-methoxyphenyl)methoxy]-2-[(3,4,5-trimethoxyphenyl)methylidene]-1-benzofuran-3-one	Phe286, Val288, Phe294, Ala296		0	
6-[2-(4-methoxyphenyl)-2-oxoethoxy]-2-[(2,4,5-trimethoxyphenyl)methylidene]-1-benzofuran-3-one	Ile263, Ile265, Leu275, Val288		0	
7-hydroxy-3-(2-methoxyphenoxy)-2-methylchromen-4-one	Ile265, Leu275, Leu276, Val288, Ser290		0	
[2-[(2,3-Dimethoxyphenyl)methylidene]-3-oxo-1-benzofuran-6-yl] 2-methylpropanoate	Gly262, Ile263, Ile265, Leu275, Val288	Gly262, Ile263	2	2.27, 2.40
3-(2-methoxyphenyl)-4-oxo-4H-chromen-7-yl dimethylcarbamate	Ile263, Ile265, Asn266, Leu275, Leu276	Ile265	1	2.50
[5-Hydroxy-3-(4-methoxyphenyl)-4-oxochromen-7-yl] 2,2-dimethylpropanoate	Ile263, Ile265, Leu275, Leu276, Val288	Ile265	1	2.20
5-hydroxy-4-oxo-2-phenyl-4H-chromen-7-yl dimethylcarbamate	Gly262, Ile263, Ile265, Leu275, Leu276, Val288	Gly262, Ile263	2	2.24,2.46
Methyl 2-{[(2Z)-2-[(3-methylphenyl)methylidene]-3-oxo-1-benzofuran-6-yl]oxy}acetate	Gly262, Ile263, Ile265, Leu275, Leu276, Val288	Gly262, Ile263	2	2.24, 2.61
(2E)-3-(2,4-dimethoxyphenyl)-1-(2,5-dimethoxyphenyl)prop-2-en-1-one	Ile263, Ile265, Leu275, Leu276, Val288		0	

Coformycin	Lys285, Glu298	Lys285, Glu298	2	2.29, 2.0
Capolactin B	Ile278, Lys285, Phe286, Lys297, Glu298	Lys285, Lys297, Glu298	4	2.83, (1.80, 2.91), 3.08
Cytosporin A	Lys297	Lys297	1	2.81
Formycin	Lys285, Phe286, Phe294, Ala296, Gln301	Lys285, Gln301	3	(2.04, 2.84), 2.23
Resveratrol	Ile278, Lys285, Phe286, Ala296, Glu298, Thr299	Lys285, Glu298, Thr299	3	2.26, 2.22, 2.24
Phenalamide A2	Ala296, Glu298	Glu298	1	2.17
Scutellarein	Asn280, Lys285, Phe286, Lys297, Glu298	Lys297, Glu298	2	2.78, 2.16
Siastatin B	Lys285, Lys297, Glu298, Thr299, Ser300	Lys297, Glu298, Thr299, Ser300	5	(3.06, 1.81), 2.61, 2.73, 2.60
Xiamycin	Ile263, Ile265, Leu276	Ile265	1	2.61
Violacein	Ile263, Ile265, Leu275, Leu276, Val288	Ile263, Ile265	3	2.37, 2.76
Sarkomycin	Thr299	Thr299	1	2.35
Anthranoside C	Ala296, Lys297, Glu298, Ser300, Gln301	Lys297, Glu298, Ser300, Gln301	5	2.92, 2.04, 2.29, (2.38, 2.71)
Apigenin	Asn280, Lys285, Phe286, Lys297, Glu298	Lys297, Glu298	2	2.77, 2.13
Baicalein	Asn280, Lys285, Phe286, Lys297, Glu298	Lys297, Glu298	2	2.78, 2.16
Pinocembrin	Ly285, Lys297, Glu298	Lys297, Glu298	2	2.72, 1.92
Rosmarinic acid	Ile278, Asn280, Lys285, Phe286, Glu298	Asn280, Lys285, Glu298	4	(2.32, 2.39), 2.58, 2.46
Noricumazole A	Ile263, Ile265, Leu276	Ile263, Ile265	3	(2.05, 2.64), 1.84

Epothilon D	Met213, Leu215, Leu225, Ile265	Leu215, Leu225	2	2.99, 2.25
Kulkenon*	Leu275, Arg279	Leu275	1	2.41
Myxochelin A	Ile265, Leu275, Val288	Ile265	1	1.80
Noricumazole C*	Thr224, Leu225, Ile227, Ala244, Leu245, Arg279	Thr224, Leu225, Ile227, Arg279	6	2.72, (2.17, 2.32, 2.67), 2.27, 2.16
Phenoxan*	Ile263, Tyr264, Ile265, Leu275, Leu276, Val288	Gly262	1	2.28
Ratjadon	Ile227, Ala244, Leu245, Ile263, Ile265		0	
Thiangazole	Ile265, Lys274, Leu275, Arg277	Lys274, Leu275	3	(1.96, 2.82), 2.84
Sulfangolid C	Glu219, Thr224, Lys274, Leu276, Ile 278	Glu219, Thr224, Lys274	4	2.81, (1.93, 2.00), 2.63
Stipiamide	Thr224, Leu225, Ile263, Ile265	Thr224, Leu225	3	(2.25, 2.63), 2.80
Soraphen F*	Leu247, Ile263, Tyr264		0	
Labindole A	Ile263, Ile265, Leu276	Ile263	1	1.94
Labindole B*	Ile263, Tyr264, Leu276	Ile263	1	1.84
3-chloro-9H-carbazole	Ile263, Ile265, Leu275, Val288	Ile263	1	9.72
4-hydroxymethyl-quinoline	Leu225, Ile227, Leu245, Ile263, Ile265	Leu275	1	2.69
Letermovir	Val185, Leu215, Glu219, Leu222, Lys226	Glu219	1	2.10
Spirangien B			0	
Capolactin A	Ile278, Lys285, Phe286, Ala296, Lys297, Glu298	Lys297, Glu298	3	2.90, (1.78, 2.97)
Aureonitol*	Leu245, Ile263, Tyr264		0	
Ganoderic acid	Ser211, Gly262	Ser211, Gly262	2	2.20, 2.99

Velutin*	Leu245, Leu247, Asp248, Tyr264	Asp248	1	1.94
Deoxyfunicone	Leu276, Lys285, Phe286, Ala296, Lys297, Glu298	Lys297, Glu298	2	2.72, 2.20
Fuscinarin	Lys285, Lys297	Lys285, Lys297	2	2.68, 2.73
Hinnuliquinone	Phe286, Glu298	Glu298	1	3.04
Griseoxanthone C	Asn280, Lys285, Phe286, Ala296, Glu298	Asn280, Glu298	3	2.05, (2.40, 2.42)
Kaempferol	Lys285, Lys297, Glu298	Lys285, Lys297, Glu298	3	2.36, 2.59, 1.92
10-methoxydihydrofuscin	Lys285, Lys297	Lys297	1	2.72
Quercetin	Ile263, Ile265, Leu275, Leu278, Val288	Ile263, Ile265	3	2.07, (2.03, 2.92)
Cytosporin A	Phe286, Phe294, Ala296, Lys297	Lys297	1	2.81
Ascorbic acid*	Arg279, Leu281, Ser282	Arg279, Leu281, Ser282	6	(2.02, 2.70,3.01), 2.71, (2.01, 2.13)
Mellein	Ile263, Ile265, Leu275, Leu276, Val288	Ile265	1	2.26
6-Hydroxymellein	Ile263, Ile265, Leu276		0	
Sabinene	Ile265, Leu275, Leu276		0	
cyclo(L-Phe-L-Pro)	Ile263, Ile265, Leu275, Leu276, Val288	Ile263	1	2.40
6-Pentyl-2H-pyran-2-one (6-Amyl-2-pyrone)	Ile263, Ile265, Leu275, Leu276, Val288		0	
Emericellin	Leu247, Leu249, Ile263, Ile265, Leu276		0	
cyclo(L-Pro-L-Val)	Ile263, Ile265, Leu275, Val288	Ile265	1	2.29
Patulin	His229, Asp239, Ala244	Asp239, Ala244	2	3.06, 3.09
Emodin*	Ile263, Ile265, Tyr264, Leu275, Leu276	Ile265, Tyr264	2	2.26, 3.58

Ergosterol peroxide	Leu247, Ile263, Ile265, Leu276, Val288		0	
Cordycepin	Asn231, His229, Asp239, Ala243	His229, Asp239, Ala243	4	2.53, (2.18, 3.58), 3.54
Curvularin*	Tyr264, Ile26, Ile265, Ile275, Ile276	Ile265	1	1.80
Citreorosein*	Arg277, Arg279, Ser288	Arg277, Arg279, Ser288	3	2.37, 2.83, 2.69
Griseofulvin*	Arg279, Leu281, Ser282	Arg279, Leu281, Ser282	4	(2.12, 3.56), 2.70, 1.90
Brefeldin A*	Gln261, Ile263, Tyr264	Gln261, Tyr264	2	2.42, 2.06
Bikaverin*	Arg277, Arg279, Ser282	Arg279, Ser282	3	2.04, (2.53, 2.67)
Xantocillin*	Ala244, Leu245, Ile227, Ile263, Tyr264, Ile265	Ile227, Tyr264	2	2.17, 2.73
Aphidicolin	Ile227, Lys274, Leu276	Ile227	2	2.23, 2.54
Cajanol	Leu225, Lys226, Ile227, His229, Ala244,	Leu225	1	2.84
Lysergic acid*	Gln261, Ile263, Ile265, Leu275, Leu276	Gln261, Ile263	2	2.83, 2.13
Ergothioneine	His229, Asp239, Ala244	His229, Asp239, Ala244	3	2.79, 2.65, 2.76
Kojic acid*	Arg279	Arg279	1	2.79
Glutathione	His229, Asn231, Asp239	His229, Asn231, Asp239	5	(2.97, 3.05), 2.18, (2.20, 2.64)
camptothecin	Gly262, Ile263, Ile265, Leu275, Leu276	Gly262, Ile263	3	(2.58, 2.70), 2.39
Tyrosol	Ile263, Ile265, Leu276		0	
Xanthone*	Ile263, Tyr264, Ile265, Leu276		0	

2,4-Diacetylphloroglucinol	His229, Asn231, Asp239	His229, Asn231, Asp239	5	(2.17, 2.36), 2.65, 2.43
Ergosterol	Leu247, Ile263, Ile265, Leu275, Leu276		0	
Ajudazol*	Ile227, Ala244, Leu245, Ile263, Ile265, Leu275, Leu276, Ile278, Arg279	Leu275, Arg279	2	2.59, 1.88
Althiomycin*	Lys226, Arg279, Asn280	His229, Arg279, Asn280	5	2.34, (1.86, 2.35, 2.70), 2.0
Angiolactone	Leu276, Ile278, Lys285, Phe286, Ala296, Lys297	Lys297	1	2.67
Aurachin E*	Ile263, Tyr264, Ile265, Leu275, Leu276, Val288		0	
Carolacton*	Ile263, Tyr264, Ile265	Ile263, Ile265	4	2.22, (2.26, 2.30, 2.38)
Chlorotonil-A	Ile263, Ile265, Leu275, Leu276, Val288		0	
Corallorazine	Ile263, Ile265, Leu275, Leu276, Val288	Ile265	1	1.97
Cystobactamid*	Ile265, Asn266, Leu275, Arg277, Arg279, Ser282	Asn266, Leu275, Arg277, Arg279	4	2.19, 2.76, 2.15, 2.52
Disciformycin A*	Gln261, Gly262, Ile263, Tyr264, Ile265, Met213, Leu215, Leu225	Gln261, Gly262, Tyr264	5	2.62, 2.49, (2.10, 2.68, 2.98)
Enhygrolide B	Gly262, Ile263, Ile265, Leu275, Leu276, Val288	Gly262	1	2.68
Hyapyrone B	Leu276, Ile278, Lys285, Phe286, Gly292, Ala296	Gly292	1	2.75
Hyalachelin	Ile263, Ile265, Leu275, Leu276, Val288, Gly292	Ile263, Gly296	2	1.77, 2.66
Hyaladione	Ly285, Lys297, Glu298	Ly285, Glu298	2	2.46, 2.39

Indiacen A	Ile278, Asn280, Lys285, Phe286, Lys297	Asn280	1	2.46
Indothiazinone	Ile278, Lys285, Phe286, Ala296, Ly297, Glu298	Ly297, Glu298	2	2.26, 2.21
Methyl indole-3-carboxylate	Lys285, Ala296, Ly297, Glu298		0	
Melithiazol A*	Gln261, Gly262, Ile263, Ile265, Leu275, Leu276, Val288	Gln261, Gly262, Ile263	4	2.20, 2.81, (1.86, 2.77)
Nannozinone A	Ile278, Asn280, Lys285, Phe286	Asn280	1	2.14
Nannozinone B*	Tyr264, Ile265, Leu275, Leu276, Val288	Ile265	1	2.22
Abietane	Ile263, Ile265, Leu275, Leu276, Val288		0	
Ambigol A	Ile265, Asn266, Leu275, Leu276, Val288	Asn266	1	2.63
Ambigol B	Ile263, Ile265, Leu276		0	
Malyngolide	Ile263, Ile265, Leu275, Leu276, Val288	Ile265	2	1.98, 2.27
Lyngbyoic acid	Ile263, Ile265, Leu275, Leu276, Val288		0	
pitinoic acid A	Ile263, Ile265, Leu275, Leu276, Val288	Ile265	1	2.09
Hapalindole	Val288, Gly292	Gly292	1	2.48
Anaephene B*	Ile263, Tyr264, Ile265, Leu275, Leu276, Val288		0	
Anaephene A	Ile263, Ile265, Leu275, Leu276, Val288		0	
Anaephene C	Ile265, Leu275, Leu276, Phe285, Val288, Phe294	Ile265	1	2.23
Cylindrofridin A	Ile265, Leu275, Val288	Ile265	1	2.03

Compounds with * interacted with the active-site residues (s) and were considered good hits.
