# Supplementary material for: In silico interaction analysis of selected natural compounds with bacteriophage-encoded hyaluronate lyase from Streptococcus pyogenes
Source: Front Med (Lausanne). 2026 Feb 16;13:1709023. doi: 10.3389/fmed.2026.1709023 (PMC12950598; doi:10.3389/fmed.2026.1709023)
Supplement: Supplementary file 2 [file Table_2.pdf]

# *Supplementary information*

# *In silico* interaction analysis of selected natural compounds with bacteriophage-encoded hyaluronate lyase from *Streptococcus pyogenes*

**Samia S. Alkhalil ***

Department of Medical Laboratory Sciences, College of Applied Medical Sciences, Shaqra University, Alquwayiyah, Riyadh, Saudi Arabia

*** Correspondence:**
Samia S. Alkhalil
salkhalil@su.edu.sa

**KEYWORDS:** bacteriophage, hyaluronate lyase, molecular docking, molecular dynamics, natural compounds, *Streptococcus pyogenes*

# *Supplementary Material*

**Table S2.** Determination of ADMET properties and assessment of Lipinski's rule of five (RO5) of the most potent compounds

| | Kulkenon | Noricumazole C | Phenoxan | Soraphen F | Labindole B | Aureonitol | Velutin | Ascorbic acid | Kojic acid | Xiamycin | SulfangolidC |
|---|---|---|---|---|---|---|---|---|---|---|---|
| LogS (logmol/L) | –4.699 | –4.011 | –5.044 | –4.223 | –2.896 | –1.887 | –3.856 | –0.482 | –0.099 | –4.634 | –3.342 |
| LogP | 3.519 | 3.124 | 4.583 | 3.774 | 2.647 | 2.068 | 3.574 | –1.814 | –0.878 | 3.592 | 2.105 |
| LogD | 3.505 | 2.567 | 4.264 | 3.548 | 2.853 | 1.729 | 2.835 | –0.599 | –0.069 | 3.251 | 2.75 |
| Pgp-substrate | No | Yes | No | Yes | Yes | No | Yes | No | No | No | No |
| PPB (%) | 75.8 | 95.780 | 97.499 | 94.827 | 92.379 | 72.348 | 93.166 | 18.822 | 53.665 | 96.6 | 71.648 |
| VD (L/kg) | 1.035 | 2.384 | 2.514 | 1.678 | 0.965 | 1.338 | 0.692 | 0.392 | 0.665 | 0.312 | –0.591 |
| BBB Penetration | No | No | No | No | Yes | Yes | No | Yes | No | No | No |
| CYP3A4 Substrate | No | No | No | No | No | No | No | No | No | No | No |
| CL (mL/min/kg) | 10.707 | 10.941 | 2.643 | 11.327 | 4.501 | 2.624 | 6.251 | 1.930 | 9.066 | 3.486 | 1.549 |
| $T_{1/2}$ | 1.172 | 0.242 | 0.235 | 0.028 | 0.809 | 0.360 | 0.701 | 0.733 | 0.888 | 1.684 | 1.427 |
| H-HT | Yes | No | Yes | No | Yes | Yes | No | No | No | Yes | Yes |
| DILI | Yes | Yes | Yes | No | Yes | No | Yes | No | No | Yes | Yes |

| | | | | | | | | | | |
|---|---|---|---|---|---|---|---|---|---|---|
| Lipinski's Rule | Accepted | Rejected | Accepted | Accepted | Accepted | Accepted | Accepted | Accepted | Accepted | Accepted | Rejected |
| Carcinogencity | No | Yes | Yes | No | Yes | No | No | No | Yes | Yes | No |
| Respiratory Toxicity | Yes | No | Yes | No | Yes | Yes | Yes | No | No | Yes | Yes |
| Eye Irritation | No | No | No | No | Yes | No | No | No | Yes | Yes | No |

PPB: Plasma protein binding; VD: Volume distribution; BBB: Blood–brain barrier; CL: Clearance; $T_{1/2}$: half-life; H-HT, human hepatotoxicity; DILI, drug-induced liver injury; LogP, logarithm of octanol/water partition coefficient; Log S, logarithm of solubility; Pgp, P-glycoprotein; LogD, logarithm of n-octanol/water distribution coefficient at pH 7.4.

**Table S2 (Continued)**

| | Emodin | Citreorosein | Griseofulvin | Brefeldin A | Bikaverin | Xantocillin | Lysergic acid | Nannozinone B | Anaephene B | violacein |
|---|---|---|---|---|---|---|---|---|---|---|
| LogS (logmol/L) | –5.594 | –3.921 | –4.447 | –3.110 | –4.731 | –3.407 | –2.806 | –3.042 | –4.157 | –4.507 |
| LogP | 3.856 | 2.661 | 2.528 | 2.139 | 3.982 | 3.070 | 0.522 | 2.327 | 4.915 | 2.688 |
| LogD | 2.845 | 1.625 | 1.628 | 1.461 | 2.376 | 3.205 | 1.306 | 2.743 | 4.219 | 2.745 |
| Pgp-substrate | No | No | No | No | No | No | No | No | No | No |
| PPB (%) | 99.295 | 94.744 | 84.889 | 78.747 | 86.456 | 78.287 | 51.220 | 95.799 | 97.011 | 98.1 |
| VD (L/kg) | 0.482 | 0.533 | 0.946 | 1.021 | 0.519 | 1.054 | 1.493 | 0.386 | 2.413 | 0.936 |
| BBB Penetration | No | No | No | Yes | No | No | Yes | No | Yes | No |
| CYP3A4 Substrate | No | No | Yes | No | No | No | No | Yes | No | No |

| | | | | | | | | | | |
|---|---|---|---|---|---|---|---|---|---|---|
| CL (mL/min/kg) | 9.245 | 7.044 | 7.740 | 15.653 | 1.312 | 7.896 | 7.908 | 9.452 | 6.153 | 2.641 |
| $T_{1/2}$ | 0.385 | 0.906 | 0.245 | 0.874 | 0.306 | 0.945 | 0.811 | 0.811 | 0.640 | 1.284 |
| H-HT | No | No | Yes | No | Yes | Yes | No | No | No | Yes |
| DILI | Yes | No | Yes | No | Yes | No | No | Yes | No | Yes |
| Lipinski Rule | Accepted | Accepted | Accepted | Accepted | Accepted | Accepted | Accepted | Accepted | Accepted | Accepted |
| Carcinogencity | No | No | Yes | Yes | Yes | Yes | No | Yes | No | Yes |
| Respiratory Toxicity | No | Yes | No | No | No | Yes | Yes | Yes | Yes | Yes |
| Eye Irritation | Yes | Yes | No | No | No | No | No | No | Yes | Yes |

PPB: Plasma protein binding; VD: Volume distribution; BBB: Blood–brain barrier; CL: Clearance; $T_{1/2}$: half-life; H-HT: human hepatotoxicity; DILI: drug-induced liver injury; LogP: logarithm of octanol/water partition coefficient; Log S: logarithm of solubility; Pgp: P-glycoprotein; LogD: logarithm of n-octanol/water distribution coefficients at pH 7.4

**Table S2 (Continued)**

| | Xanthone | Ajudazol | Althiomycin | Aurachin E | Carolacton | Cystobactamid | Disciformycin A | Melithiazol A | Chlorotonil-A |
|---|---|---|---|---|---|---|---|---|---|
| LogS (logmol/L) | –4.005 | –4.705 | –2.089 | –2.569 | –4.246 | –4.137 | –3.246 | –5.172 | –5.176 |
| LogP | 3.317 | 5.015 | 0.830 | 7.559 | 3.142 | 4.265 | 3.051 | 4.405 | 4.088 |
| LogD | 2.893 | 4.554 | 1.038 | 4.856 | 2.120 | 2.369 | 2.094 | 4.149 | 3.722 |
| Pgp-substrate | No | No | Yes | No | No | Yes | Yes | No | No |
| PPB (%) | 92.230 | 99.070 | 66.032 | 86.262 | 94.961 | 99.396 | 79.543 | 98.069 | 97.3 |
| VD (L/kg) | 0.500 | 1.002 | 0.672 | 4.770 | 0.278 | 0.337 | 1.611 | 0.329 | 1.393 |
| BBB Penetration | No | No | Yes | No | No | No | No | No | No |

| | | | | | | | | | |
|---|---|---|---|---|---|---|---|---|---|
| CYP3A4 Substrate | No | No | No | No | No | No | No | Yes | No |
| CL (mL/min/kg) | 1.960 | 6.773 | 1.600 | 4.285 | 8.557 | 0.899 | 6.564 | 3.278 | 3.474 |
| $T_{1/2}$ | 0.224 | 0.303 | 0.208 | 0.125 | 0.608 | 0.624 | 0.330 | 0.041 | 1.129 |
| H-HT | No | No | No | Yes | Yes | No | Yes | Yes | No |
| DILI | Yes | No | Yes | No | Yes | Yes | Yes | Yes | Yes |
| Lipinski Rule | Accepted | Rejected | Accepted | Accepted | Accepted | Rejected | Accepted | Accepted | Accepted |
| Carcinogenicity | Yes | Yes | No | Yes | No | No | No | No | Yes |
| Respiratory Toxicity | Yes | Yes | Yes | No | No | No | No | No | Yes |
| Eye Irritation | Yes | No | No | No | No | No | No | No | Yes |

PPB: Plasma protein binding; VD: Volume distribution; BBB: Blood–brain barrier; CL: Clearance; $T_{1/2}$: half-life; H-HT: human hepatotoxicity; DILI: drug-induced liver injury; LogP: logarithm of octanol/water partition coefficient; Log S: logarithm of solubility; Pgp: P-glycoprotein; LogD: logarithm of n-octanol/water distribution coefficients at pH 7.4
